# Lowered inter-stimulus discriminability hurts incremental contributions to learning

**DOI:** 10.3758/s13415-023-01104-5

**Published:** 2023-09-01

**Authors:** Aspen H. Yoo, Haley Keglovits, Anne G. E. Collins

**Affiliations:** 1 Department of Psychology, University of California, Berkeley, USA; 2 Helen Wills Neuroscience Institute, University of California, Berkeley, USA; 3 Department of Cognitive, Linguistic and Psychological Sciences, Brown University, Providence, USA

**Keywords:** Reinforcement learning, Working memory, Computational modeling

## Abstract

How does the similarity between stimuli affect our ability to learn appropriate response associations for them? In typical laboratory experiments learning is investigated under somewhat ideal circumstances, where stimuli are easily discriminable. This is not representative of most real-life learning, where overlapping “stimuli” can result in different “rewards” and may be learned simultaneously (e.g., you may learn over repeated interactions that a specific dog is friendly, but that a very similar looking one isn’t). With two experiments, we test how humans learn in three stimulus conditions: one “best case” condition in which stimuli have idealized and highly discriminable visual and semantic representations, and two in which stimuli have overlapping representations, making them less discriminable. We find that, unsurprisingly, decreasing stimuli discriminability decreases performance. We develop computational models to test different hypotheses about how reinforcement learning (RL) and working memory (WM) processes are affected by different stimulus conditions. Our results replicate earlier studies demonstrating the importance of both processes to capture behavior. However, our results extend previous studies by demonstrating that RL, and not WM, is affected by stimulus distinctness: people learn slower and have higher across-stimulus value confusion at decision when stimuli are more similar to each other. These results illustrate strong effects of stimulus type on learning and demonstrate the importance of considering parallel contributions of different cognitive processes when studying behavior.

**Supplementary Information:**

The online version contains supplementary material available at 10.3758/s13415-023-01104-5.

## Introduction

Humans are efficient learners but how fast we learn depends heavily on what we learn about. For example, a teacher learning the name of two new transfer students may only need to be told their names once, but they may need much more trial and error for each student if they’re learning the name of the entire class at the same time. Furthermore, if the students look alike, learning may require even more effort. Here, we formally explore how stimulus discriminability (in a semantic and visual domain) impacts learning, and whether the multiple processes involved in learning are affected differently.

Specifically, we investigate stimulus discriminability in a stimulus-action association task in which both reinforcement learning (RL) and working memory (WM) processes are utilized (e.g., Collins & Frank, 2012). Reinforcement learning (RL) broadly refers to the process that characterizes how people learn incrementally through valenced feedback (Sutton & Barto, 1998). Working memory (WM) is a flexible, but capacity-limited process involved in actively maintaining perceptually unavailable information over a short period of time (Cowan, 2017). While there has been an increase in investigating the interplay between these two essential processes (for a review, see Yoo & Collins, 2022), there still is much to be learned about how the two interact in different settings.

For example, researchers in both RL and WM fields consider stimulus carefully when designing experiments, but each field tends to focus on different aspects of stimuli. RL studies tend to use a variety of stimuli across tasks. Sometimes they use stimuli with low semantic information, such as gabor patches, fractals, and foreign alphabet characters (e.g., Farashahi et al., 2017; Niv et al., 2015; Oemisch et al., 2019; Wilson & Niv, 2012; Wunderlich et al., 2011; Radulescu et al., 2019; Daw et al., 2011), under the assumption that relying on stimuli that are easy to name and have high semantic discriminability (i.e., have different names), such as different common objects, shapes, and colors (Collins & Frank, 2012; Collins, 2018; Farashahi et al., 2020), may affect behavior (perhaps by employing more explicit processes like WM). WM studies’ choice of stimuli is much more explicit, due to traditional WM being formalized as being modality specific (i.e., containing separate visual and verbal storage units; Baddeley & Hitch, 1974). Stimuli that are nameable (e.g., spoken words, digits, or words) are considered to relate to verbal WM (e.g., Conrad, 1964), while less easily nameable stimuli (e.g., orientations, spatial frequencies) correspond to visual WM (e.g., Luck & Vogel, 1997; Wilken & Ma, 2004).

From previous research, it is apparent that there is some consideration of how different stimuli may affect behavior. However, it is still unclear how stimulus discriminability affects RL, WM, or their interplay. How do different types of stimuli affect RL and WM processes during an associative learning task? Specifically, are RL and WM differently affected by how distinct stimuli are? To address our question, we designed and collected data on two stimulus-response association learning experiments, manipulating stimulus discriminability. Learning was measured in three stimulus conditions.

There is evidence that human learning differs for abstract and naturalistic stimuli (Farashahi et al., 2020), so one of our primary criteria when choosing stimulus sets was for them to be similarly “naturalistic” and similarly familiar (vs. novel). Our first condition, the “Standard” condition, we used a standard stimulus set, in which the stimuli images that were discriminable visually and semantically. Second, the “Text” condition had stimuli which were simply text printed of different nouns, designed to limit visual information while maintaining semantic information. Finally, in our “Variants” condition, stimulus sets contained different example images of the same noun, designed to decrease semantic discriminability across stimuli without simplifying the stimuli themselves (i.e., images alone had full semantic information, but as a group caused interference by all being associated with the same name). We investigated the effect of these conditions through behavioral comparisons of learning behavior across the three conditions and two load conditions, as well as computational modeling to try to understand changes in the underlying RL and WM processes across conditions.

Generally, we predicted that both RL and WM would be necessary to capture behavior in all conditions, but that the processes would behave differently across the three stimulus conditions. However, due to 1) the fact that both Text and Variants conditions likely had lowered discriminability in both visual and semantic dimensions and 2) the potentially competing effects between RL and WM, it was difficult to predict exactly how changes in RL, WM, and their interplay would affect the ultimate behavioral performance across conditions. Take, for example, the Variants condition vs. the Standard condition. An assumption in the RL literature is that learning associations from stimuli with semantic information (e.g., Standard condition) may recruit “more explicit” processes like WM, and thus that a Variants condition could avoid contamination from explicit processes and better access to implicit learning ones. However, the assumption that decreasing semantic discriminability would lower the contribution of WM in learning is untested. In fact, the visual WM literature consistently demonstrates that WM representations need not be verbalizable at all. Additionally, people are able to reliably discriminate between WM representations of naturalistic stimuli with the same label (Brady et al., 2016). Similarly, if RL is indeed an implicit process, as often hinted in the literature, then stimulus condition should not impact it much. However, if RL instead relies heavily on distinct semantic information across stimuli, performance should suffer in the Variants condition. Thus, while we had a strong prediction that stimulus type would impact learning, and could impact the different processes supporting learning in different ways, we did not have a strong prediction as to the exact nature of this impact. We designed the study with an eye to behavioral modeling to help understand the intertwined processes.

**Fig. 1:**
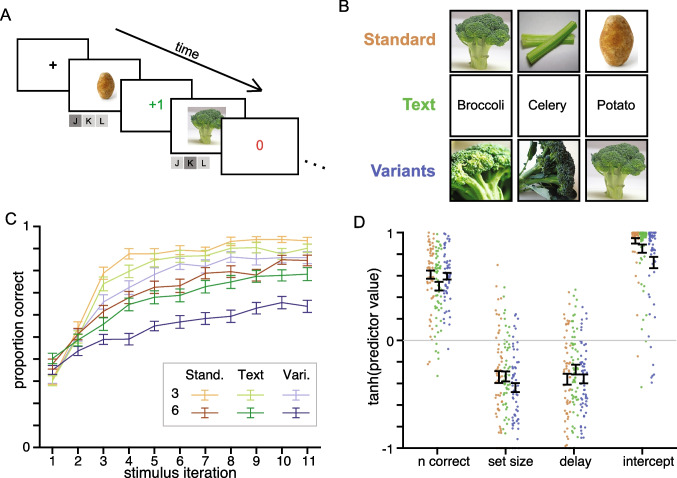
Experiment 1 task and learning curves. A. Behavioral task. Participants learn through trial and error, with veridical, deterministic feedback, the correct response to each stimulus. B. Example “vegetable” stimuli, for the three different stimulus conditions: Standard, Text, Variants. Stimulus categories were different for each block, so participants would never see (for example) a broccoli in multiple learning blocks. C. Learning curves ($M \pm SEM$ over participants) show the proportion of correct choices as a function of the number of times a stimulus has been encountered within a block (stimulus iteration), for each stimulus condition (color) and set size (value/saturation). While 11 stimulus iterations are illustrated, some stimuli were presented more times

Our results confirmed that stimulus type impacted learning; we observed lower performance in the Variants and Text conditions relative to the Standard condition, demonstrating that overall discriminability is important in learning. The behavioral deficit was particularly pronounced in the Variants condition. Through computational modeling, we found that stimulus conditions seemed to specifically affect RL, and not WM.

## Experiment 1

In Experiment 1, participants completed a Conditional Associative Learning paradigm, learning correct stimulus-action associations through feedback.

### Experimental Methods

#### Participants

Eighty-eight participants were recruited through Amazon Mechanical Turk (MTurk), provided informed and written consent, and verified they were adults. The study was in accordance with the Declaration of Helsinki and was approved by the Institutional Review Board of University of California, Berkeley (IRB 2016-01-0820). Participants received $0.50 base payment for participating, and earned bonus payments for the time they spent on the task and their accuracy. Participants were informed that each correct response would increase their payment, and were reminded of this when starting each block. On average, participants made $3.30 and spent 42 minutes on the task. Participants who were performing below chance after the fourth or eighth block were discontinued from completing the task, but were compensated for their time. Participants who performed under 40% accuracy overall were additionally excluded from further analyses. 19 participants did not complete the task and 10 participants did not meet the accuracy threshold, leaving 59 participants in the final online sample.

#### Experimental design

Participants completed a Conditional Associative Learning paradigm (Petrides, 1985), adapted to investigate the contributions of RL and WM in learning (Collins & Frank, 2012; Collins et al., 2014). At the beginning of each block, participants viewed a screen that displayed the set of stimuli that would be used on that block. They were instructed that each stimulus had a single correct button press associated with it, and that their goal was to learn the correct association using trial-and-error. On each trial in the block, participants viewed a centrally-presented stimulus from this set and had up to 1500 milliseconds to press one of three buttons on a keyboard to respond (Fig. 1a). Participants received binary, deterministic reward feedback after each response indicating whether the response was correct for this stimulus. If participants failed to respond within 1500ms, the screen indicated “response too slow,” and were coded as nonresponses for subsequent analyses. Each stimulus was presented approximately 13 times within a block (stimuli were presented as few as 11 and as many as 14 times). Participants learned sets of either 3 or 6 images (stimuli) at a time, resulting in two set sizes for analysis. The larger set size (6 stimuli) resulted in greater WM load as well as longer delay times between repetitions of the same stimulus, and thus were more difficult. Because all stimuli were presented approximately the same number of times, the total number of trials per block was either 39 or 78. All blocks had the same number of keypress options (3), and the information about any stimulus-key pairing was not informative of any others within or across blocks (i.e., it was not the case in the 3 stimuli blocks that each stimulus mapped to a different key). Thus, chance performance was 33%.

In addition to the set size condition, each block also belonged to one of the three following stimulus conditions (Fig. 1b):

- Standard: stimuli are images of different subcategory members belonging to the same category (e.g., vegetables: broccoli, celery, potato), and easily discriminable both semantically and visually.
- Text: stimuli are words printed in black letters on a white background, corresponding to subcategory name (e.g., the words “broccoli,” “celery,” “potato”). This condition is designed to provide full editcolor semantic information as Standard, but lowered visual discriminability within stimulus set.
- Variants: stimuli are different images of the same subcategory (e.g., different images of broccoli). This condition is designed to provide rich visual information, but limited distinct semantic information relative to the Standard condition – each image within a set was designed to call to mind the same word to limit the ability to have unique verbal labels for each image.

One of our primary criteria for choosing the stimuli across conditions was for them to be similarly naturalistic and familiar/recognizable to the participants. There is evidence that humans learn differently between abstract and naturalistic stimuli (Farashahi et al., 2020). Furthermore, differences in familiarity could also impact learning. Stimuli in the Standard condition were based on prior studies using the RLWM design (Collins & Frank, 2012), and were taken from ImageNet, a crowdsourced dataset commonly used to train the computer vision networks on image classification.

Variants condition images were also acquired from ImageNet, but chosen to call to mind the same word. Based on reported verbal strategies from prior studies using RLWM tasks, we predicted that allowing for extraneous visual variance could lead to alternative labeling strategies (for example, labeling a broccoli on a farm “farm” and a broccoli on a kitchen table as “table”), so we additionally minimized the possibility of additional distinguishing features (e.g., all images of broccoli on a plain background). While there is less visual discriminability in the Variants condition than the Standard one, the images are certainly not perceptually confusable, for they vary along lower-level visual dimensions (e.g., broccoli in different orientations, of different size, shades of green). Ultimately to keep stimuli naturalistic, we opted to use images that alone, had full semantic information (i.e., were individually nameable), but as a group caused interference (i.e., were all associated with the same name).

With similar motivation, we chose to use Text for a condition that had full semantic information while limiting visual information. While it would have been ideal to use images that looked alike but depicted different things, we could not think of such visual stimuli while satisfying the naturalistic and familiar constraints we imposed on our stimulus conditions. We thus compromised by simply writing the words out (i.e., showing a picture of black letters on a white screen), lowering visual information overall without sacrificing semantic information.

Each block had a unique category (e.g., vegetables, farm animals, clothing items), so a participant would not see, for example, stimuli corresponding to “farm animals” in both the Standard and Variants conditions. Which category was assigned to each stimulus condition, and what order they were presented in, was counterbalanced across participants, so participants saw different subsets of the entire stimulus set. The block order of the set size and stimulus conditions were also pseudorandomized across participants. Participants completed two blocks per set size x stimulus condition as well as one practice and one final block, completing a total of 780 trials over 14 blocks. We did not consider the first and last block in any analyses to remove potential effects of practice or fatigue, leaving 702 trials for analysis.

### Experimental Results

Learning was successful in all conditions, indicated by an increasing proportion of correct responses as a function of stimulus iteration (Fig. 1c). As in prior studies using the RLWM design, participants responded slower in the set size 6 blocks than in the set size 3 blocks. However, a two-way repeated measures ANOVA with stimulus condition, set size, and their interaction showed that while the difference between the set sizes was significant ($p<.001$), there was no effect of stimulus condition ($p=.62$) on reaction time, nor an interaction between condition and set size ($p=.57$). Reaction times are not analyzed further, but are shown in Supplementary Fig. S1. To describe experimental effects on accuracy, we conducted a two-way repeated-measures ANOVA with stimulus condition, set size, and their interaction as independent variables, as well as separate intercept terms for each participant. There was a significant effect of set size, such that set size 3 blocks had overall better mean performance ($M=.79$, $SEM=.02$) than set size 6 blocks ($M=.66$, $SEM=.02, F(1,58) = 106.2, p<.001$, Fig. 1c), supporting the involvement of WM in learning and replicating prior work using this paradigm (e.g., Collins, 2018). There was a significant main effect of condition ($F(2,116) = 43.95, p <.001$), such that performance in the Variants condition ($M=.66, SEM =.02$) was significantly lower than both Standard ($M=.78, SEM=.02, p<.001$) and Text conditions ($M=.74,SEM=.02, p<.001$). Standard and Text conditions were not significantly different ($p=.18$). The p-values for posthoc tests are Bonferroni corrected. Finally, there was a significant interaction between condition and set size ($F(2,116) = 6.803, p=.002$); this was due to a stronger effect of condition in set size 6 ($F(2,116) = 38.8, p<.001$) than set size 3 blocks ($F(2,116) = 8.71, p <.001$). This suggests that stimuli differences are more critical for learning when learning more stimulus-action associations simultaneously.

While the ANOVA reveals gross overall effects, it neglects the progress of learning across set sizes and conditions; to better qualify this experimental effect we conducted a logistic regression. For each participant and condition, we investigated whether we can predict trial-by-trial accuracy based on the previous number of correct outcomes for that stimulus, the set size, and the delay since last correct. We found results consistent with previously reported studies (e.g., Collins & Frank, 2012; Collins et al., 2014), such that the probability of a correct response on the current trial was positively related to previous number of correct (as expected from incremental RL-like learning), and negatively related to set size and delay in all conditions (as expected from WM contributions to learning; predictors are illustrated in Fig. 1d).

### Modeling methods

While descriptive statistics allow us to qualify the effects of set size and learning for each condition, these tests do not allow us to understand how the underlying processes, RL and WM, produce these behavioral differences across conditions. For this, we turn to behavioral modeling. Like previous publications using similar tasks and models (e.g., Collins & Frank, 2012; Viejo et al., 2015; Jafarpour et al., 2022), we assume participants’ responses depend on both RL and WM processes. We describe the general “RLWM” framework, then consider different models that make different condition-specific predictions.

#### General model formulation

In this section, we describe the building blocks of the models we will be testing. We describe the basic learning rules for the RL and WM processes and how a policy is derived from each process’s representation of stimulus-action associations.

**Learning rules** In this section, we discuss the learning rules for the RL and WM processes. We refer to the stimulus (s) action (a) value pairs as Q-value for RL process, *Q*(*s*, *a*), as is standard in the model free reinforcement learning literature, and the corresponding stimulus-action association pairs for WM process as WM, $\textrm{WM}(s,a)$. When we refer to operations that apply to both functions interchangeably, we generalize using the term “value function”, which we denote *V*(*s*, *a*).

*RL learning rule.* This is the classic Rescorla-Wagner model, in which the observer iteratively learns the value of each stimulus-action response through trial-and-error feedback. After observing reward $r_t$, the participant updates the Q-value as follows:

$$\begin{aligned} \forall s,a \hspace{2pt} Q_0(s,a)&= \frac{1}{N_a} \nonumber \\ Q_{t+1}(s,a)&\xleftarrow []{} Q_t(s,a) + \alpha (r_{t+1}-Q_t(s,a)), \end{aligned}$$

where $N_a$ is the number of possible actions (3 in our experiment) and $\alpha$ is the learning parameter. The larger $\alpha$, the more informative the current trial is in the Q-value. To allow for learning asymmetry (e.g., Frank et al., 2007; Niv et al., 2012; Gershman, 2015; Sugawara & Katahira, 2021), we use two different learning rates for positive (correct) and negative (incorrect) rewards. We fit models in which both $\alpha$ and $\alpha _-$ are free parameters, as well models in which $\alpha _-$ is fixed to 0 (Xia et al. , 2021; Eckstein et al. , 2022). In the main manuscript, we report only the models in which $\alpha _-=0$, for relaxing this assumption did not improve model fit and did not change the main results or conclusions (Supplementary S1.7.2).

*WM learning rule.* The WM observer updates the association value of stimulus-action pairs immediately to the observed reward, but this “perfect” information is subject to memory decay. The value association update is as follows:

$$\begin{aligned} \forall s,a \ \text {WM}_0(s,a)&= \frac{1}{N_a} \\ \text {WM}_{t+1}(s,a)&\xleftarrow []{} r_{t+1}, \end{aligned}$$

for $r=1$, which can be thought of as a Rescorla-Wagner update rule with an $\alpha = 1$ and $\alpha _-=0$. The WM decay is implemented by, on every trial, having all stimulus-action associations decay towards their starting value:

$$\begin{aligned} \forall s,a \ \ \text {WM}_{t+1}(s,a) \xleftarrow {} (1-\lambda ) \text {WM}_{t+1}(s,a) + \lambda \text {WM}_0(s,a), \end{aligned}$$

where $\lambda$ is the decay rate. With this formulation, WM’s stored values regress to uninformative values, $\text {WM}_0(s,a)$, for items that have been seen longer ago.

**Calculating response probability.** We assume that the observer chooses action $a_i$ with probability based on a softmax function:

$$\begin{aligned} p_V(a_{i}|s) = \frac{e^{\beta V_t(s,a_{i})}}{\sum _{i=1}^3 e^{\beta V_t(s,a_{i})}}, \end{aligned}$$

where $\beta$ is the inverse temperature parameter and controls the stochasticity in choice, with higher values leading to a more deterministic choice of the best value action. Here, we fix $\beta$ to an arbitrarily high number, 100. Fixing $\beta$ to a high number enforces behavior we find to be a necessary theoretical baseline: it simulates behavior that is true to the way WM is theorized (it enforces close to perfect one-back WM policy under low load) whilst still being consistent with the general formulation of RL models. Additionally, it is common practice in “RLWM” models (e.g., Jafarpour et al., 2022; McDougle & Collins, 2020), and improves interpretability of parameters (i.e., parameter recovery is only successful when $\beta$ is fixed). $V_t(s,a_i)$ depends on the given state *s*, action $a_i$, and process (RL vs. WM).

*Perseveration.* Models with perservation incorporate the tendency of agents to respond based on previous actions, irrespective of the current stimulus and reward (e.g., Sugawara & Katahira, 2021).

$$\begin{aligned} V_t(s,a_i) = V_t(s,a_i) + \phi C_t(a_i), \end{aligned}$$

where $\phi$ denotes how strongly a participant perseverates in their responses, and $C_t(a_i)$ is the choice trace vector of action $a_i$. The models in the main text define $C_t(a_i) = 1$ if the choice on trial $t-1$ was $a_i$, and 0 otherwise. (We fit all models without perseveration, and fits were significantly worse across models. We additionally allow perseveration choice to be affected by trials more than one trial back, with decay parameter $\tau$; this addition does not approve the fits. Details can be found in Supplementary S1.7.3).

**Response policy.** The probability of responding action $a_i$ given state *s*, $p(a_i|s)$ is a weighted sum of the contribution from the RL and WM process.

$$\begin{aligned} p(a_i,s) = \omega _np_\text {WM}(a_i|s) + (1-\omega _n)p_\text {RL}(a_i|s), \end{aligned}$$

where the mixture weight $\omega _n$ is a value between 0 and 1, corresponding to the WM contribution for blocks with set size *n*. In a fully RL-driven model, $\omega _n=0$; in a fully WM-driven model, $\omega _n = 1$. We predict that $\omega _6 < \omega _3$ because there is lower WM contribution in higher set size conditions, but we do not impose this constraint during model fitting.

*Random responses.* We additionally assume that, with proportion $\epsilon$, participants randomly choose an action. We are agnostic to whether this behavior reflects a response lapse, a random guess, or greedy exploration. The final response policy at time *t*, $\pi _t$ is thus

$$\begin{aligned} \pi _t(a_{i}|s) = (1-\epsilon )p(a_{i}|s) + \frac{\epsilon }{N_a}. \end{aligned}$$

#### Models

In this section, we describe the six models we considered. All models assume that both RL and WM are involved in the learning process, but make different assumptions about whether and how each of the two processes are affected by stimulus conditions. We did not consider models in which only RL or only WM are involved, for neither would be able to capture data across set sizes, let alone across conditions (Supplementary Fig. S17). First, we test three models in which RL process is affected specifically. We test one model in which condition-differences in learning are assumed to be a result of different learning rates (RL learning rate). We test alternative models that assume confusion *within* a stimulus set results in noisier learning: either that updating the current stimulus accidentally updates other stimuli in the same block (RL credit assignment), or that retrieving the values of the current stimulus is confused with other stimuli (RL decision confusion). Second, we consider two models in which the WM process is affected specifically, either through differing decay (WM decay) or decision confusion (WM decision confusion) across conditions. Finally, we consider a model that assumes that the RL and WM processes aren’t changed in isolation based on stimulus condition, but the interaction between the two (RL WM weight). This model hypothesizes that the observer relies on RL and WM to different degrees, depending on stimulus condition. Alternative assumptions, different specifications for perseveration or nonzero negative learning rate $\alpha _-$ are presented in Supplementary Materials S1.7, but these did not better explain our data than the models presented here.

**Condition-specific RL learning rate.** Motivated by the observation that stimulus condition influences accuracy, we first consider a model which assumes that stimulus condition impacts how quickly RL updates Q-values. We implement this assumption by fitting three separate $\alpha$ parameters, one for each stimulus condition. We denote the learning parameter for Standard, Text, and Variants stimuli as $\alpha _s$, $\alpha _t$, and $\alpha _v$, respectively.

**Condition-specific RL credit assignment.** In the “RL credit assignment” observer, we test the assumption that the lowered performance in different conditions is not due to lowered learning rates, but increased difficulty to distinguish the stimuli which leads to credit assignment confusion. Credit assignment confusion occurs when updating Q values not only for the current trial’s stimulus, but also for other stimuli, leading to potential future interference between stimuli. For example, when a reward is obtained for a given choice and stimulus, the rewarded choice would also be credited to other stimuli, although those stimuli may require a different correct action.

With standard RL and WM learning rules, the observer only updates state-action values for the current stimulus, $s_i$. With credit assignment confusion, all other stimuli in the current block (which are not relevant to the current trial) are also updated to a lesser degree, parameterized by weight $0\le \eta \le 1$:

$$\begin{aligned} \forall s_j \ne s_i: V_{t+1}(s_j,a) \xleftarrow []{} V_t(s_j,a) + \alpha \eta (r_{t+1}-V_t(s_i,a)). \end{aligned}$$

We fit credit assignment confusion parameters to Text and Variants conditions only, denoted $\eta _t$ and $\eta _v$, respectively. We did attempt to fit a model with credit assignment confusion in the Standard condition, $\eta _s$, and did not include in the main manuscript because parameter recovery was not successful for that model; this is likely because a combination of other parameters (e.g., $\alpha$, $\beta$, $\lambda$, $\epsilon$) can characterize noise in a way that is behaviorally difficult to distinguish from credit assignment alone. In this sense, we assume that any credit assignment confusion in the Standard condition would be generally captured by noise parameters, and that the **additional** confusion in the Text and Variants conditions would be captured by the condition-specific parameters. This additional confusion is our primary interest, for we are interested in the difference in performance across conditions.

**Condition-specific RL decision confusion.** In the “RL decision confusion” observer, we test the assumption that the lowered performance in different conditions is due to across-stimulus decision confusion when the observer is calculating their response policy. In other words, the confusion is not in the encoding of the state-action values (like the RL credit assignment model), but the retrieval of values when making a decision. Decision confusion is implemented during the decision stage, such that all stimuli in the current block that are not relevant to the current trial are also used to calculate the response policy for the RL process:

1 $$\begin{aligned} V'_t(s,a_{i}) = (1-\zeta )V_t(s,a_{i})+\zeta \frac{1}{N_s-1}\left( \sum _{\lnot s} V_t(\lnot s,a_i)\right) , \end{aligned}$$

where $N_s$ is number of stimuli, parameter $\zeta$ is a scalar between 0 and 1, and indicates how much across-stimulus decision confusion there is. A value of 0 indicates no decision confusion, and a value of 1 would indicate full confusion. We fit decision confusion parameters for the Text and Variants conditions, denoted $\zeta _t$ and $\zeta _v$, respectively. Like in the RL credit assignment model, we implicitly assume there is no RL decision confusion in the Standard condition, $\zeta _s=0$, for modeling parsimony and recoverability, or that RL decision confusion is absorbed by other noise in that condition. In that sense, again, this model assumes additional processes in the Text and Variants conditions, to attempt to capture observed performance drops.

**Condition-specific WM decay** In this model, we test the assumption that WM decay is solely responsible for performance differences across conditions. Rather than learning the values faster in certain conditions, we just remember the associations better. We denote the WM decay for Standard, Text, and Variants stimuli as $\lambda _s$, $\lambda _t$, and $\lambda _v$, respectively.

**Condition-specific WM decision confusion** This model is the WM analog to the RL decision confusion model. In this model, we test the assumption that participants have across-stimulus decision confusion when calculating the response policy for the WM process, according to Eq. 1.

**Condition-specific weight** In this model, we test the assumption that different weights between the RL and WM processes results in different behavior, rather than condition differences resulting from changes in either process. So, when encountering different stimuli, either system could be modulated to have a larger or smaller effect. In this model, the weights $\omega$s differ across condition and set size, and are denoted with subscript. For example, $\omega _{6s}$ corresponds to the RLWM weight of a set size 6 Standard stimulus condition. We include the simplifying assumption that the differences across conditions in set size 3 blocks are minimal, and use $\omega _3$ for all set size 3 stimulus conditions. Thus, the Condition-specific weight model has four $\omega$ parameters, $\omega _3, \omega _{6s}, \omega _{6t},$ and $\omega _{6v}$.

#### Parameters and estimation

The parameters for each model, $\theta$ are displayed in Table 1. All models we consider contain the following fitted base parameters: RL learning rules with positive learning rate $\alpha$, WM with forgetting rate $\lambda$, perseveration with proportion $\phi$, response policies which are a weighted combination of RL and WM components with a weighted sum (determined by weight $\omega _3$ and $\omega _6$ for set size 3 and 6, respectively), and random responses with proportion $\epsilon$. Model-specific parameters are presented in the, aptly named, “Model-specific parameters” column.

For each participant and each model, we maximized the logarithm of the likelihood (*LL*) of the data given the parameters and model $\log (p(\text {data}|\theta ))$, using fmincon in MATLAB with 20 random starting points. The largest *LL*, $LL^*$, and the associated parameter $\theta$ are assumed to be the global maximum-likelihood parameter estimates.

**Table 1:** **Model parameters**. Free parameters for each model. Base parameters are loosely comparable across all models; model-specific parameters are additional ones fit to capture condition-specific effects

Model	Base parameters	Model-specific parameters
RL learning rate	$\alpha _s, \lambda , \phi , \omega _3, \omega _6, \epsilon$	$\alpha _t,\alpha _v$
RL credit assignment	$\alpha , \lambda , \phi , \omega _3, \omega _6, \epsilon$	$\eta _t, \eta _v$
RL decision confusion	$\alpha , \lambda , \phi , \omega _3, \omega _6, \epsilon$	$\zeta _t, \zeta _v$
WM decay	$\alpha , \lambda _s, \phi , \omega _3, \omega _6, \epsilon$	$\lambda _t,\lambda _v$
WM decision confusion	$\alpha , \lambda , \phi , \omega _3, \omega _6, \epsilon$	$\zeta _t, \zeta _v$
RL WM weight	$\alpha , \lambda , \phi , \omega _3, \omega _{6s}, \epsilon$	$\omega _{6t}, \omega _{6v}$

#### Model and parameter recovery

A crucial, but often overlooked, step in interpreting model parameters and in quantitative model comparison is making sure parameter values are meaningful and that models are identifiable (Nilsson et al., 2011; Palminteri et al., 2017; Wilson & Collins, 2019). In order to establish the interpretability of model parameters, one should test that the same parameters that generate a data set are the ones estimated through the model parameter estimation method. Successful parameter recovery exists when one is able to “recover” the same (or similar) parameter values that generated the data.

Successful model recovery is an important step for making conclusions from quantitative model comparisons. Successful model recovery occurs when the same model that generates a data set is the model that best fits it (according to your chosen model comparison metrics), when compared to all other models in the comparison set. We obtained reasonable parameter recovery and model recovery; details and figures for both analyses are in Supplementary Sections S1.4 and S1.5).

#### Model comparison

Because all of our models have 8 parameters, we report model goodness-of-fit by simply comparing $LL^*$, the maximum LL across all runs for a participant and model. In addition to $LL^*$, we compared fits across participants with group Bayesian Model Selection (BMS; Stephan et al., 2009; Rigoux et al., 2014). While summed $LL^*$ assumes all participants are generated by the same model, BMS explicitly assumes that participants can be best fit by different models. BMS assumes that the distribution of models is fixed but unknown across the population, and uses the log marginal likelihoods for each model and participant to infer the probability of each model across the group. This method is sensitive to both the distribution and magnitude of the differences in log-evidence. From this, we can compute the protected exceedance probability (*pxp*), which is how likely a given model is to be more frequent than the other models in the comparison set, above and beyond chance. A lower summed $LL^*$ and higher *pxp* indicate better model fit to data.

### Modeling Results

**Fig. 2:**
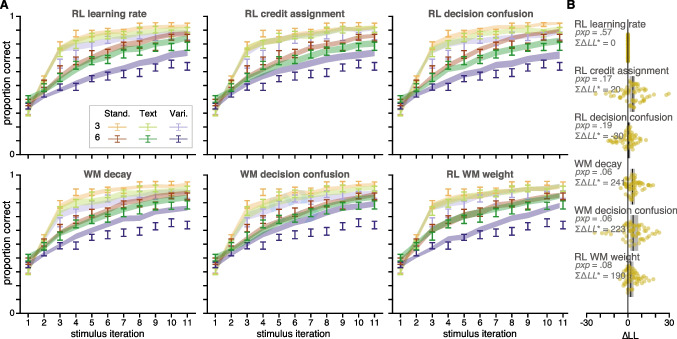
**Experiment 1 Modeling Results.** A. Learning curves for each condition (color) and set size (value/saturation) across participants for data (errorbars, $M \pm SEM$) and model predictions (fills, $\pm SEM$). Only the first 11 stimulus iterations are illustrated, but all iterations were used in modeling. B. Difference in LL scores for each model, relative to the RL learning rate model. Dots indicate individual participants, black line indicates median, and grey box indicates 95% bootstrapped confidence interval of the median. Difference of summed $\Delta LL^*$s across participants and protected exceedance probability displayed for each model. Lower $LL^*$s and higher *pxp*s indicate better model fit

Both metrics gave similar results, favoring the RL learning rate model over the RL credit assignment, WM decay, WM decision confusion, and RL WM weight models. The RL decision confusion model performed similarly well to the RL learning rate model. We illustrate individual-participant, median $\Delta LL^*$s, summed $\Delta LL^*$s, and *pxp*s in Fig. 2b.

Second, we qualitatively compared the models’ ability to generate data similar to that of the real data. For example, posterior predictive checks are an important step in assessing model fits, particularly for data with sequential trial dependencies (Palminteri et al., 2017); a simple model of the weather that predicts today’s weather is the same as yesterday’s may result in high likelihoods without being able to actually predict weather patterns. For each participant, we simulated data using the MLE parameters for each participant, and find that the qualitative fits to the data (Fig. 2a) reflect the quantitative model comparison; the models that feature either condition-specific RL learning rates or condition-specific RL decision confusion provide a better fit to the true data than other models. These results suggests that different stimulus conditions affect exclusively the RL process, by how efficiently it learns from or uses reward information.

### Interim conclusions

In Experiment 1, we asked how limiting discriminability in editcolor semantic or visual information across stimuli changes people’s ability to learn stimulus-response associations in a load-dependent RL task. First, we replicated the set size effect, showing that for all task conditions a load of 6 stimuli produced worse performance than blocks with only 3 stimuli, indicating WM’s role in task performance. Second, and to our main question, we found that limiting either discriminable visual or semantic information across stimuli detrimented performance. This condition effect interacted with load such that it had a larger effect in higher load conditions, suggesting that the condition may tax the RL system that is more responsible for behavior in the larger load conditions.

We used computational modeling to investigate if we could explain the process by which this performance detriment occurs, and found that a model that either assumes that people have lower RL learning rates or have higher confusion across stimuli when calculating the RL response policy was able to capture the data reasonably well qualitatively, and quantitatively better than other models. However, all models predict slightly higher performance in the Variants condition set size 6 relative to human performance (Fig. 2). In Experiment 2, we designed an experiment to more directly test the contribution of RL in learning, by adding a surprise memory test.

## Experiment 2

**Fig. 3:**
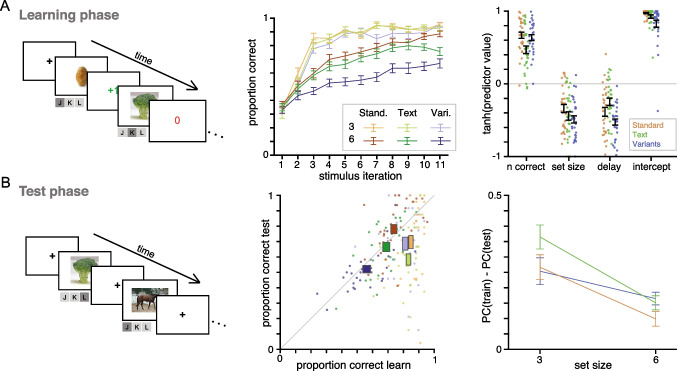
**Experiment 2 task and results.** A. Learning phase. *Left*: Task design. *Middle*: Proportion of correct choices increases as a function of stimulus iteration for all stimulus and set size conditions but slower for set size 6, especially in the Variants condition. *Right*: Logistic regression. For all three conditions, participants are more likely to select the correct response when it is a lower set size block, shorter delay, and when they have gotten more correct responses on that stimulus previously. B. Test phase. *Left*: task design. Participants viewed all stimuli previously learned and reported their believed correct response. No correctness feedback was given. *Middle*: Proportion correct in training (x-axis) and testing (y-axis) phase for condition (color), showing individual participants (dots) or $M \pm SEM$ across participants (boxes). *Right*: Tortoise and hare effect: there is a larger deficit in long-term retention (difference in proportion correct (PC) from train to test) with stimuli learned in set size 3 blocks than set size 6 blocks. This deficit was not significantly different across conditions

Our second experiment was designed to replicate and extend the behavioral and modeling results of the first experiment. First, participants completed the same stimulus-response paradigm as in Experiment 1. Participants then completed a “Test phase”, after a WM distractor task, designed to clear WM. During the Test phase, all stimuli from all Learning phase blocks were presented again in random order, and participants responded which of the three response keys they believed to be the correct response. No feedback on correctness was given. This phase probed how well stimulus-response pairs were learned by a RL process, presumably without the aid of WM.

### Experimental Methods

#### Participants

Thirty-seven participants (22 female, mean age 21) were recruited through a UC Berkeley online site and received course credit for experimental participation. Participants in this experiment did not receive any bonus compensation based on performance. We obtained informed, written consent from all participants. The study was in accordance with the Declaration of Helsinki and was approved by the Institutional Review Board of University of California, Berkeley (IRB 2016-01-0820). Seven participants were excluded for psychiatric diagnosis disqualifications, withdrawing early, not being fluent in English, or monitor malfunctions in the testing rooms, leaving 30 (19 female, mean age 21) participants in the final online sample.

#### Experimental design

Participants completed the same stimulus-response learning paradigm, with the same numbers of trials and blocks, as in Experiment 1. In addition to this “Learning Phase”, participants additionally completed a WM distractor task and a “Test Phase”, which they were not told about ahead of time.

In the distractor task, participants completed 5 blocks of a N-back task. This task was designed to tax the WM system, clearing any working memory information about stimulus-response mappings from the Learning Phase, and is not analyzed in main manuscript. More details about this task can be found in the Supplementary Materials Section S1.2. It took approximately 10 minutes to complete.

Lastly, participants completed a surprise Test Phase, in which all stimuli from the Learning phase blocks were presented again in random order. Because the Test phase was beyond both WM capacity (54 associations tested) and maintenance period for most stimuli, this phase probed how well stimulus-response pairs were learned by a RL process alone. For each trial, a stimulus was presented, participants responded which of the three response keys they believed to be the correct response, and no feedback on correctness was given. Each of the 54 unique stimuli from the learning block was presented four times, for a total of 216 trials. Only stimuli from the middle 12 blocks (i.e., excluding stimuli from the first and last block) were included in this test phase to limit primacy or recency effects of memory (Murdock Jr., 1962). Because each Learning phase block corresponded to a unique category (i.e., a participant would see stimuli corresponding to “vegetables” in only one stimulus condition), there should not be any category-specific interference between blocks. All trials were completed in a single block.

### Experimental Results

Here, we analyze the behavioral results from the Learning phase and Test phase. First, we analyze learning phase data as done in Experiment 1 (Fig. 3a, middle). We conducted the repeated measures ANOVA, with proportion correct as the dependent variable and set size and stimulus condition as independent variables. There was a significant effect of set size ($F(1,29) = 185.1$, $p<.001$), condition ($F(2,58) = 24.66$, $p<.001$), and interaction between set size and condition ($F(2,58) = 11.90$, $p <.001$). For condition, performance in the Variants condition ($M=.69, SEM=.03$) was significantly lower than that of the Standard ($M=.79, SEM = .02, p<.001$) and Text ($M=.76,SEM=.02,p=.02$) conditions. Performance was not significantly different for Standard and Text conditions $p=.53$). The interaction was driven by a nonsignificant condition effect in set size 3 blocks ($F(2,58) = 2.44$, $p = .10$) but a strong condition effect in set size 6 blocks ($F(2,58)= 27.07$, $p<.001$). We then conducted the logistic regression to test whether the likelihood of responding correctly on the current trial could be predicted from the previous number correct for that stimulus, the set size, and the delay since last correct. We found results consistent to Experiment 1 such that the probability of getting a correct response on the current trial was positively related to previous number of correct, and negatively related to set size and delay (Fig. 3a, right). Reaction time analyses revealed the same pattern of results as in Experiment 1: participants responded slower in the set size 6 blocks than in the set size 3 blocks, but an ANOVA showed that while the difference between the set sizes was significant ($p<.001$), there was no effect of stimulus condition ($p=.11$) or an interaction between condition and set size ($p=.80$; Supplementary Fig. S1).

Second, we analyzed the participants’ performance on the Test phase. Collins and others (2018) demonstrated an interaction between RL and WM processes for long-term retention of the correct stimulus-action pair. Items in lower set size blocks had better performance during the Learning phase compared to higher set size blocks, but interestingly, a larger detriment in performance in the Test phase. This “tortoise and hare” effect demonstrated a trade off between RL and WM process; while WM assists performance during learning, it detriments long-term retention of the stimulus-action pairs. For all conditions and set sizes, performance was above chance ($t(29) > 6.35, p<.001$), suggesting long-term retention of stimulus-response associations even without explicit instruction to do so. Second, there was a significant positive correlation across participants between the proportion correct in the Learning and Test phases ($r = .40, p=.03$). Finally, the difference between performance in Learning phase and Test phase was much larger in trials corresponding to stimuli learned in set size 3 blocks than ones learned in set size 6 blocks ($t(29) = 6.41, p<.001$), replicating the tortoise and hare effect, showing interference of WM with RL learning. We conducted a one-way repeated measures ANOVA and found no statistical difference in the magnitude of this “tortoise and hare” effect across conditions ($F(2,58) = 2.207, p = .12$). This nonsignificance of magnitude of deficit suggests that the difference in WM used between set size 3 and 6 in each condition is nonsignificantly different.

### Modeling methods

#### Replication of Experiment 1

We first analyzed the Learning phase of Experiment 2 identically to that of Experiment 1. Details on the six models, fitting procedure, and model comparison can be found in Section the modeling section above.

#### Investigating Test phase

We additionally investigate model fit by jointly fitting Learning and Test phase data. In other words, all data are used to calculate the likelihood of parameter given model parameters and data. The likelihood of learning phase data are computed identically to the previous procedure. For test phase data, we assume that participants only have access to RL values, not WM association weights; thus the likelihood of test phase trials relies only on the Q-values learned during the learning phase, which are frozen through the test phase in absence of feedback (Collins, 2018). *LL*s are optimized in the same way as Experiment 1, and model are compared in the same way as Experiment 1. We fit the two best fitting models: the condition-specific RL learning rate and condition-specific RL decision confusion models.

**Fig. 4:**
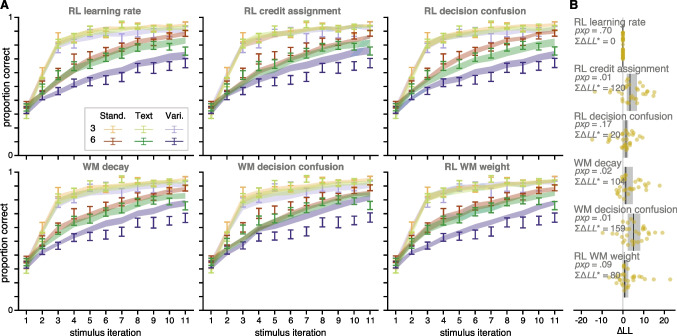
**Experiment 2 modeling results: replication of experiment 1** A. Learning curves for each condition (legend at top) across participants for data (errorbars, $M \pm SEM$) and model predictions (fills, $M \pm SEM$). Only the first 11 stimulus iterations are illustrated, but all iterations were used in modeling. B. Difference in $LL^*$ for each model relative to the RL learning rate model. Dots indicate individual participants, black line indicates median, and grey box indicates 95% bootstrapped confidence interval of the median. Difference of summed $LL^*$s across participants and protected exceedance probability displayed for each model. Lower $LL^*$s and higher *pxp*s indicate better model fit

We additionally test, for the RL learning rate and RL decision confusion models, the assumption that RL and WM processes are not independently updating value in during the learning phase, but actually interact during learning. As in Collins (2018), we implement this assumption such that WM contributes cooperatively during learning when calculating the RPE used by the RL process:

2 $$\begin{aligned} \delta _t = r_t - (\omega _n WM_t(s,a) + (1-\omega _n)Q_t(s,a)). \end{aligned}$$

We refer to this set of model as models “with interaction” (e.g., RL learning rate model with this modification is the “RL learning rate + interaction” model).

For all models, we additionally fit a softmax inverse temperature parameter, $\beta$, for the Test phase, under the assumption that response noise in using RL Q-values will likely differ for each participant between Training and Test phase due to failures in long-term retention of stimulus-response associations.

### Modeling Results

We modeled the data in Experiment 2 in two ways. First, we fit only the Learning phase data, as in Experiment 1, to see if we could replicate those results. Second, we jointly fitted parameters on Learning and Test phase data, to see if modeling results differed from results when only fitting Training phase data.

**Replication of Experiment 1** Modeling results were remarkably consistent with Experiment 1; the condition-specific RL learning rate model fit the substantially better than most models across participants, and similarly as well as the RL decision confusion model. These two models were best able to produce model predictions that looked qualitatively similar to that of the actual data (Fig. 4a). They were additionally able to capture the data quantitatively the best (Fig. 4b).

**Fig. 5:**
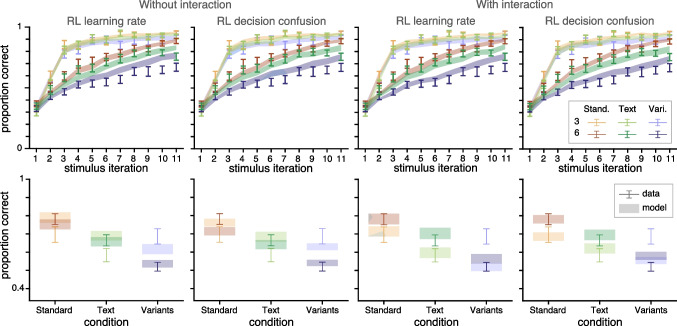
**Exp 2 learning and test phase model validation**. Model validation for RL learning rate and RL decision confusion models without (left two plots) and with (right two plots) an interaction between RL and WM processes during learning. Model predictions (fill) and data (error bars) for models jointly fitted on Training (top) and Test phase (bottom) data

**Investigating Test Phase** Model validation plots are illustrated in Fig. 5. Quantitatively, model performance was very similar (lower summed $\Delta LL^*$ and higher *pxp* indicates better model fits to data). RL learning rate summed $\Delta LL^* = 0$, $pxp=.25$; RL decision confusion summed $\Delta LL^* = 49$, $pxp=.23$; RL learning rate + interaction summed $\Delta LL^* = -44$, $pxp=.27$; RL decision confusion + interaction summed $\Delta LL^* = -8$, $pxp=.25$).

Qualitatively, the models that assume an interaction between RL and WM during learning were able to capture Test phase data better for the Standard and Text condition (orange and green), but models that assume no interaction were able to capture Test phase data better in the Variants condition (blue). As a follow up, we considered models that had condition-specific interaction strengths, but they were not able to fit the data substantially better than those reported here (Supplementary S1.7.5).

## Further model investigations

### Interpreting model parameters

We investigated the parameter values for the two best-fitting models: the condition-specific RL learning rate and the condition-specific RL decision confusion models (individual and group parameter values for models fit on Learning phase displayed in Supplementary S1.6).

We first investigated whether it was reasonable to combine participants across the two experiments, for the models that were fitted to only Learning phase data. For each model, we conducted Welch’s t-tests for each parameter with a Bonferroni correction across parameters. We found for both winning models, no parameters were significantly different across experiments ($p>.41$).

For all following analyses, we combine participant parameters across experiments.

To investigate the differences between condition-specific parameters for each the model, we conducted Wilcoxon signed-rank test with a Bonferroni correction across the number of pairwise tests. First, we investigated whether the learning rates, $\alpha$s, across conditions differ in the condition-specific RL learning rate model. The learning rate for Variants condition ($\alpha _v$: $M =.01$, $SEM = .003$) was significantly lower than that of Text condition ($\alpha _t$: $M=.03$, $SEM= .006$, $z=-7.40$, $p<.001$) and Standard condition ($\alpha _s$: $M=.04$, $SEM=.008$, $z=-6.37$, $p<.001$). The difference in learning rates for Standard and Text condition were not statistically significant ($z=2.25$, $p=.07$). For the models fit to both Learning and Test phase data in Experiment 2, the results are largely consistent, finding that learning rate for the Variants (no interaction model: $M=.01, SEM=.001$, interaction model: $M=.008, SEM=.0008$) condition is lower than that of Standard (no interaction: $M=.04, SEM=.03,z=-4.37, p <.001$; interaction: $M=.04,SEM=.02,z=4.41,p<.001$) and Text (no interaction: $M=.01,SEM=.003, z=-2.99, p=.008$; interaction: $M=.02,SEM=.004$,$z=3.38,p=.002$) conditions. However, models that were fitted on both phases also found a statistically significant difference between Text and Standard conditions (no interaction: $z=2.77, p=.02$; interaction: $z=2.79, p=.02$).

For the RL decision confusion model, we found that the decision confusion for the Variants condition ($\zeta _v$: $M=.44$, $SEM =.02$) was significantly higher than that of the Text condition ($\zeta _t$: $M =.22$, $SEM=.03$, $z = 6.02$, $p <.001$). This effect is also true for the models fitted on Learning and Test phase of Experiment 2; decision confusion is greater in the Variants condition than the Text condition in both the models that assume no interaction between RL and WM (Variants: $M=.36, SEM=.04$, Text: $M=.18, SEM=.04$, $z=2.95, p=.003$) and those that do (Variants: $M=.40, SEM=.04$, Text: $M=.20, SEM=.04$, $z=3.38, p=.001$).

### Alternative models

As in all modeling papers, we cannot possibly sample all possible models of this data. In our final analysis, we test two additional models that embody more complex hypotheses, as a control. We fit just the Learning phase data, and do not assume any interaction between RL and WM during learning.

**Table 2:** Experiment 1 quantitative model comparison

	RL learning rate	RL decision confusion	WM decay	RL learning rate + WM decay	Superfree
*pxp*	.21	.01	.00	.00	.77
exp$_r$	.31	.18	.04	.08	.39
mean($\Delta$AICc)	0	-1	8	0	-4
med($\Delta$AICc)	0	1	7	1	2

Protected exceedance probability (*pxp*), expected posterior probabilities (exp$_r$), mean AICc differences relative to RL learning rate (mean($\Delta$AICc)), and median AICc difference (med($\Delta$AICc)). Positive AICc values indicate that RL learning rate provides a better fit to the data

**Condition-specific RL learning rate and WM decay** Our previous models assumed that only one process was affected by stimulus condition. In this model, we test the assumption that both processes are affected. To minimize additional complexity, we consider the model that lets the two most likely parameters from each process be condition dependent; specifically, this model assumes that RL learning rate and WM decay both depend on stimulus condition. Theoretically, this model allows us to test the assumption that both processes may differently but jointly contribute to differences in behavior. This model has the following 10 parameters $\alpha _s, \alpha _v, \alpha _t, \lambda _s, \lambda _v, \lambda _t, \phi , \omega _3, \omega _6, \epsilon$.

**Superfree** The “Superfree” model fits each condition entirely separately. Thus, it is extremely unconstrained, overparameterized, and lacks theoretical justification on its own. However, it provides a *qualitative* upper bound for the explainability of all models considered in this paper. We consider this model an important metric to use when considering the goodness-of-fit of models during model validation. This model has a total of 21 parameters, consisting of 7 parameters for each condition: $\alpha , \lambda , \phi , \zeta , \omega _3, \omega _6, \epsilon$.

#### Model comparison and results

For model comparison with the new additions, we focus on the previous winning models, as well as the previous best candidate model where WM parameters were condition dependent. Specifically, we select 1) RL learning rate and 2) RL decision confusion, and 3) the WM decay model. Because the models considered in this section have different numbers of parameters, we use corrected Akaike Information Criterion (AICc; Hurvich & Tsai, 1987) to quantitatively compare model goodness-of-fit. Like AIC (Akaike, 1972), AICc penalizes models with more parameters, using parameters as a proxy for model flexibility (and additionally corrects for potentially low trial numbers):

$$\begin{aligned} \text {AICc}&= -2LL^*+2k +\frac{2k(k+1)}{N_\text {trials}-k-1} \end{aligned}$$

where *k* is the number of parameter and $N_\text {trials}$ is the number of trials. We chose to use AICc verses other model comparison metrics, because it provided us the best model recoverability, although it penalizes parameters less strictly than Bayesian Information Criterion (BIC). We report the median and mean of the difference between the AICc of one model and the RL learning rate model ($\Delta$AICc); larger values provide larger support in favor of the RL learning rate model. In addition to reporting the protected exceedance probability of each model *pxp*, we report the expected posterior probability of each model, denoted exp$_r$. These two metrics provide us a more heterogeneous interpretation of model goodness-of-fit, such that different models may be superior for different subsets of participants. All quantitative results for Experiment 1 and 2 are reported in Tables 2 and 3, respectively.

**Table 3:** Experiment 2 quantitative model comparison

	RL learning rate	RL decision confusion	WM decays	RL learning rate + WM decay	Superfree
*pxp*	.30	.04	.04	.05	.56
exp$_r$	.33	.09	.04	.14	.39
mean($\Delta$AICc)	0	1	7	1	-1
med($\Delta$AICc)	0	3	3	2	0

Protected exceedance probability (*pxp*), expected posterior probabilities (exp$_r$), mean AICc differences relative to RL learning rate (mean($\Delta$AICc)), and median AICc difference (med($\Delta$AICc)). Positive AICc values indicate that RL learning rate provides a better fit to the data

Our results in this section are consistent with our other modeling results, for both experiments and for all model comparison metrics. First, as shown previously, both RL-only models individually fit better than the WM-only models in both experiments. Second, they individually fit better than the new model that assumed both RL and WM were affected by stimulus condition, suggesting that assuming condition-dependent WM changes does not provide any additional explanatory power to assuming only RL is affected (though, results of model recovery may weaken the interpretation of this result; Figs. S13, S14) . Third, the model that assumed both RL and WM were both affected fit better than the WM-only model, suggesting that condition-specific RL modulation is key to fitting human behavioral data.

Interestingly, the RL-only models are not favored over the Superfree model in either experiment. These quantitative results do not reflect a simple overfitting; the Superfree model is not the best fitting model for data simulated by other models (i.e., model recovery is successful for our chosen model comparison metrics. Figure S13), and is qualitatively superior at capturing behavior in the set size 6, Variants condition (Fig. S20). While the Superfree model seems to be capturing *some aspects* of behavior that others model are not, the overparameterization of the model (indicated by poor parameter recovery, Fig. S11) makes it difficult to understand, in a meaningful way, why. On the other hand, the RL learning rate model still provides a superior fit for a nontrivial proportion of participants (Experiment 1 / 2: exp$_r$ = .31 / .33), suggesting that it is a competitive model, whilst still being interpretable.

## Discussion

In this study, we investigated how the type of information across a stimulus set affected learning. Participants learned the correct response to stimuli that had different levels of discriminability relative to other stimuli in the same block. In behavior across two experiments, we show that,when there are more items to learn about concurrently, performance suffers minimally in the Text condition relative to the Standard condition, but substantially in the Variants condition.

Through computational modeling, we found that the differences in learning behavior across stimulus conditions were driven by deficits in specifically the RL process. The models that best predicted behavior was the one that either assumed that, across conditions, the RL learning rate changed or that there was confusion in the RL system at the decision stage. These models fit better than those that assumed stimulus condition affected credit assignment in RL, WM decay, decision confusion in WM, or the weight between RL and WM. Additionally, models that assumed the RL was alone affected fit better than a model that assumed both RL and WM were affected by stimulus condition.

What could be causing the differences in learning across the two lowered-discriminability stimulus conditions? Perhaps there is a preference for the modality of stimulus. Perhaps the deficit in the Variants condition was driven by a lack of semantic distinctness. Many RL studies actively select non-nameable stimuli with the (often implicit) goals of targeting putatively implicit processes (Frank at al., 2004; Daw et al., 2011) and limiting the contributions of other, more explicit cognitive processes. Consequently, they rely on the hypothesis that stimulus information in the semantic domain may impact learning, and in particular the balance of RL processes and higher level processes such as inference or memory. In contrast to that interpretation, our results suggest that the semantic distinguishability of the stimuli affects RL itself, not a different process and not its interaction with another process.

Our results are consistent with that of Radulescu and others (2022), who more directly tested nameability of stimuli on learning. Like us, they found that more nameable stimuli were associated with higher RL learning rates, and that the effect of nameability on performance was more apparent in larger set size conditions. This interpretation is consistent with the results in the Text condition as well. Because stimuli were still semantically discriminable, performance on the Text stimulus condition was not significantly worse than that of the Standard stimulus condition.

In contrast to the RL process, our computational results suggest a lack of impact of stimulus condition on the WM process. Perhaps this is due to sufficient information being available to WM regardless of stimulus condition. Let’s consider the Variants condition, in which a lack of semantically distinct information across stimuli does not hurt learning behavior. In other words, there was sufficient visual information between stimuli that WM processing was not affected. This explanation seems feasible given the research on WM for visual stimuli. The visual WM literature has demonstrated that, despite WM being information-constrained, people are able to learn and prioritize information in WM that is most relevant to performance (Yoo et al., 2018; Bays, 2014; Klyszejko et al., 2014; Emrich et al., 2017; Sims, 2015), even when stimuli are extremely simple and non-verbalizable (e.g., oriented lines, dots in space). Perhaps prioritization of relevant information would be easier with naturalistic stimuli; WM performance for naturalistic stimuli demonstrated to be better than with simple stimuli (Brady et al., 2016), and even more so for objects familiar to participants (Starr et al., 2020, even when doing a simultaneous verbal task, to ensure verbal WM is not assisting). Our results and this literature together suggest that, unlike RL, WM can learn actions associated with a stimulus set with low semantic discriminability, as long as there is high visual discriminability (and vice versa). In other words, WM is able to discriminate stimuli and maintain stimulus-response associations equally well with only visual or semantic information. It is important to note, though, that while we designed these stimulus sets with visual and semantic modalities in mind, we did not quantify the difference between discriminability across conditions. Thus, it is possible that our interpretation of how visual vs. semantic information affects processing may be overly simplified.

What other processes could be causing the differences in learning in the RL process across stimulus conditions, beyond a simple modality preference? It is known that learning a category structure becomes more difficult with increased similarity of exemplars between categories (Love et al., 2004; Nosofsky, 1986) and increasing number of dimensions required to distinguish categories (Nosofsky et al., 1994; Shepard et al., 1961). This difficulty is apparent in the Variants condition, in which participants had to distinguish between stimuli based on relatively low-level visual differences that are not often of ecological importance. This is in contrast to the Text condition, in which stimuli are so easily discriminable due to the association of the word with its meaning – a relatively automatic association, as seen in the well-replicated Stroop task (1935) – despite having relatively similar low-level visual characteristics across stimuli. In the Variants condition, unlike the Text condition, what features were important to pay attention to itself became something that needed to be learned (Leong et al., 2017), and likely affected behavior. For example, “learning traps” can occur in behavior (Rich & Gureckis, 2018), due to selective attention, simplification, or dimensionality reduction (Nosofsky et al., 1994; Goodman et al., 2008). The poor performance in the Variants condition could have been because the relevant discriminating features in the Variants condition (e.g., luminosity, absolute size, orientation of object) are, in the other two experimental conditions and often in real life, trivial compared to object identity – your value assessment for an apple doesn’t depend on how bright the room is. The combination of interference (due to interleaved condition blocks) and a learning trap (previous experience within and beyond the experiment indicating these low-level features are unimportant) could have resulted in difficulty successfully using these features to discriminate between stimuli for RL. Other studies corroborate this conclusion, finding stimulus type (e.g., naturalistic stimuli learned better than abstract stimuli; Farashahi et al., 2020) and response “state” (e.g., motor responses learned better than stimulus responses; Rmus & Collins, 2020) affect learning. Regardless of exact cognitive mechanism at play, these results demonstrate the importance of considering how a learning state is defined.

Our results have strong implications for understanding the neural circuits that support flexible learning. Previous research has focused on clarifying how the brain integrates past choice and reward history to make a choice given a stimulus, with little consideration to the inputs of this computation - such as the stimuli. Past findings have shown that multiple distinct neural systems contribute to learning. Reinforcement learning computations appear to be implemented in cortico-basal ganglia loops (Alexander et al., 1986; Haber, 2011; Collins & Frank, 2014), with striatum playing a crucial role in supporting iterative, reward-dependent learning (e.g., McClure et al., 2003; O’Doherty et al., 2003; Frank et al., 2004; Frank & O’Reilly, 2006). Prefrontal cortex activity also reflects reward prediction errors in feedback-based learning tasks (e.g., Barto, 1995; Schultz et al., 1997; Shohamy et al., 2004; Daw et al., 2011), but is typically thought to be more related to flexible goal-directed behavior (e.g., Hampton et al., 2006; Valentin et al., 2007). Specifically, there has been evidence that PFC function supports WM in the context of learning, in parallel to subcortical RL (Collins & Frank, 2012; Collins et al., 2017). While there is a growing understanding of the multiple neural mechanisms that support learning, and in particular the RL circuits in the brain, the inputs to this network are not often carefully considered - RL computations assume known stimuli, actions, and rewards as inputs to learn a policy (Rmus et al., 2021). Here, our work shows that the inputs, in particular the state space, matter: the nature of the stimuli impacted RL computations, slowing learning and potentially increasing choice confusion. It would be interesting in future research to do network-level modeling to understand how this behavior may arise from more diffuse/overlapping input representations.

Neuroscientific research in RL contrasts with that of WM, which has spent a considerable amount of effort investigating how stimulus information affects WM representations in the brain. Namely, neuroscientific research has demonstrated that WM in the brain is highly distributed, and that the brain areas involved vary depending on the type of information being maintained (for review, see Christophel et al., 2017). For example, in addition to the prefrontal cortex, retinotopic maps in occipital and parietal cortices are related to the WM maintenance of visual information (Harrison & Tong, 2009; Riggall & Postle, 2012). However, despite neural WM representations being represented through sensory cortices, WM still behaves similarly in the context of learning and decision making, where the conjunction of stimuli and correct choices is the most important information to be maintained. Perhaps this associative, higher-level information is successfully represented in the PFC, regardless of specific stimulus information. Future research with brain imaging could shed more light on this.

There are, of course, limitations to our results. First, while our model fits are reasonable, there are still some qualitative deviations in our model validation and the data we collected. In particular, learning performance in the Variants condition in set size 6 was lower than the RL learning rate model predictions. Perhaps learning detriments in the Variants condition is a combination of other, unconsidered processes interacting with either RL or WM. There has been ample research that computationally, behaviorally, and neurologically demonstrate that other processes interact with RL and/or WM. For example, episodic memory interacts with memoranda maintained in WM (e.g., Hoskin et al., 2019) and choice in RL tasks (e.g., Bornstein & Norman, 2017). Attention also affects both WM (e.g., Chun et al., 2011; Souza et al., 2018) and RL (e.g., Farashahi et al., 2017; Leong et al., 2017; Niv et al., 2015). While it would be lovely to be able to study all these processes in tandem, it is simply out of the scope of this project; the design of our experiment would likely not allow different processes to be distinguished behaviorally or computationally.

Second, and more critically, we were not able to conclusively distinguish whether it was lower learning rate or increased across-stimulus confusion during the RL response policy calculation. Perhaps the experimental design is too simple to distinguish the choice noise that occur from both cases. However, these “RL learning rate” and “RL decision confusion” models are distinguishable according to model recovery (Supplementary S1.5), so it is not simply that they make similar predictions. Additionally, these results do not suggest just a simple increase in noise, since other models that also result in increased behavioral noise (i.e., RL credit assignment, WM decay, and WM decision confusion models) do not fit the data quantitatively or qualitatively as well. Thus, our results do strongly suggest an impact on *specifically* the RL process. Understanding the exact nature of that impact will require additional study, likely with different paradigms.

Our two experiments were conducted in fairly different demographics and experimental environments: Experiment 1 was conducted online on MTurk and Experiment 2 was conducted in person in an undergraduate population. Despite subtle differences in behavior across the two experiments (namely, the difference in statistical significance of condition differences in set size 3 blocks), we find remarkable consistency in behavior, model rankings, qualitative goodness of fits of winning models, and estimated parameters across experiments. Thus, we see the two experiments as a broad replication of results as a sign of robustness of the findings.

Overall, this study replicates results demonstrating the importance of both RL and WM in the study of learning. This study provides evidence that stimulus matters in learning, potentially pointing to the importance of semantic information in learning. We find an interesting result that condition differences only affected the RL process, while the WM process was largely spared. This paper strongly demonstrates the importance of considering how a learning state is defined. Future research should continue to investigate how different stimuli/states affect learning and, at the very least, consider how the experimental choice of stimuli affects learning behavior.

### Supplementary Information

Below is the link to the electronic supplementary material.

Supplementary file 1 (pdf 9420 KB)

## Data Availability

Participant and simulated data are available at https://osf.io/f4hst/. Plotting and analysis code are available at https://github.com/aspenyoo/RLWM_stim_discrim. None of the experiments were preregistered.
