# Supplementary material for: Lowered inter-stimulus discriminability hurts incremental contributions to learning
Source: Cogn Affect Behav Neurosci. 2023 Sep 1;23(5):1346–64. doi: 10.3758/s13415-023-01104-5 (PMC10545593; doi:10.3758/s13415-023-01104-5)
Supplement: Supplementary file 1 — (pdf 9420 KB) [file 13415_2023_1104_MOESM1_ESM.pdf]

S1 Supplementary Materials

In the Supplementary Materials, we include additional analyses that broadly support the main text. We include details on participant reaction times on the Learning phase, N-back distractor task, qualitative differences in error types between the two winning models, parameter recovery, model recovery, and alternative models that were tested. In the alternative models, we included analyses of RL, WM, and RLWM models; whether model goodness-of-fit changes with a fixed or fitted perseveration rate and negative learning rate; and whether perseveration choice trace is greater than one trial back.

S1.1 Reaction times

Plotted below are the individual subject (dots) and group mean (bars) reaction times in seconds, split by stimulus condition and set size.

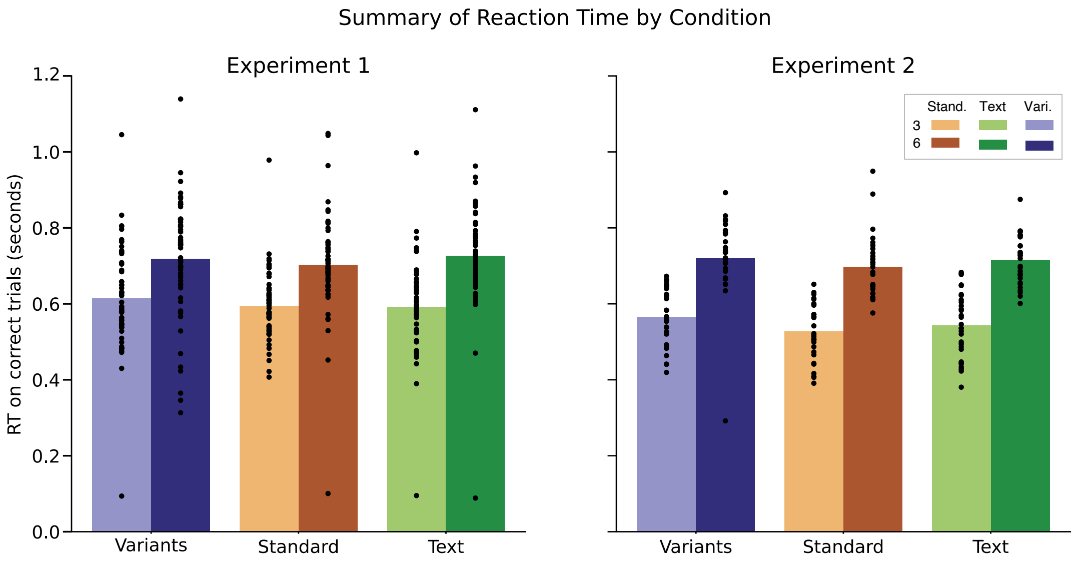

Figure S1: **Subject Reaction Times by Experiment.** Mean (bar) and individual participant (dots) reaction times for each condition, for the learning phase of Experiment 1 (left) and Experiment 2 (right). Reaction times were not used as a means of exclusion for either experiment.

S1.2 N-back distractor task

The first block was a practice block with N=2, then the following four blocks incrementally increased from N=2 to N=5. Each block had on average 40 trials, and the stimulus shown on each trial was a colored rectangle; potential rectangle colors were common and distinct from one another (e.g., blue, yellow, pink, black, green). Code for the N-back task can be found at https://github.com/AlexanderFengler/ExperimentDesign_NBackTask.

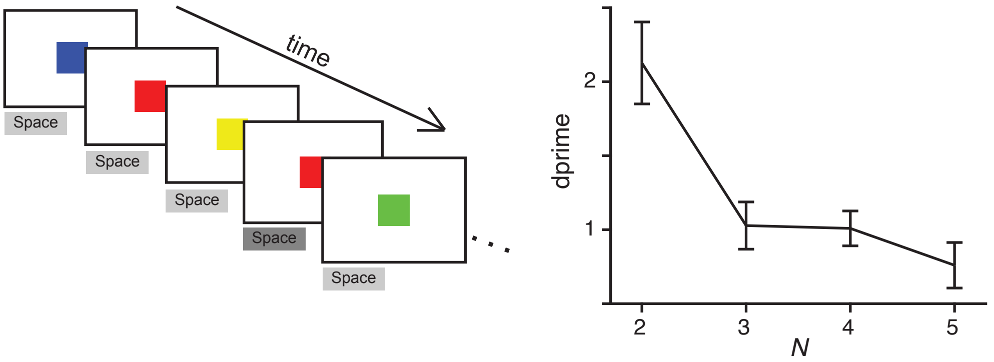

Figure S2: N-back task. *Left*: task design. Participants viewed a series of colors and made a key press every time the color N trials ago was the same as the color of the current block. This illustration demonstrates all correct responses on a $N = 2$ back task. *Right*: d' decreases a function of N, indicating worse performance with increasing set size.

S1.3 Qualitative difference between models: error types

We found that the models that assumed that either there was a condition-specific effect on RL learning rate or a condition-specific effect on RL decision confusion were able to fit the data best. While the goal of our paper is not to find one model that explains all datasets we collected, it is still an interesting question to ask what the differences are between participants best fit by each of the models. In this section, we highlight one qualitative difference between the two winning models.

To investigate qualitative differences between models, we analyzed the key press errors. Unlike learning curves, the two models *should* generate different predictions on error types. For the RL learning rate model, errors are primarily driven by a lower rate of learning, so errors should be randomly distributed across incorrect keys. On the other hand, if people are confusing stimuli at the decision stage, errors should not be random. Specifically, the RL decision confusion model should predict that errors would be skewed toward the key presses that are rewarded in other stimuli.

For all set size 3 blocks, there was an imposed structure such that there was a key for which two images were correct, a key for which one image was correct, and a key for which no images were correct. (The correct keys were counterbalanced across blocks.) Because the correct answers were not evenly distributed across key presses, we were able to investigate if errors are random or reflect the distribution of correct keys across all trials (i.e., independent of current stimulus). We cannot do this analysis on set size 6 blocks, since each key had 2 images each associated with it.

For each participant, we split up errors by whether the correct answer was the key that was correct for two stimuli (which we will refer as the "2" key) or if the correct answer was correct for

only one stimulus (the "1" key). We then calculated the proportion of the incorrect key presses that were correct for a different stimulus (incorrectly pushing the "1" or "2" key), versus a key that was never rewarded (the "0" key). If errors are random, as predicted by the RL learning rate model, this proportion would be around 0.5. If errors result from decision confusion, participants' error should be biased toward stimuli rewarded in other trials. However, there are other reasons that decisions would be biased toward stimuli rewarded in other trials (e.g., a general avoidance of never-rewarded key). If errors are truly a result of decision confusion, there should be higher confusion in trials in which 1 is correct but 2 is pushed, than trials in which 2 is correct but 1 is pushed.

For visualization, we grouped the participants by whether they were better fit by the RL learning rate or RL decision confusion model (i.e., which model had a higher $LL*$). In Experiment 1, 35 participants were best fit by the RL learning rate model, and 24 best fit by the RL decision confusion model. In Experiment 2, 19 participants were best fit by the RL learning rate model, and 11 best fit by the RL decision confusion model. Proportion of error types for both Learning and Test phase are illustrated in Figure S3.

For both phases, we conducted a two-way ANOVA for each group of participants, to investigate whether the error types were different according to condition (Standard, Text, Variants), correct key (2 or 1), and interaction between the condition and correct key. For the RL learning rate group, in both Learning and Test phase, we found no significant main effect of condition, correct key press, and no significant interaction. Preference for key rewarded in other trials in Learning ($t(53) = 7.30, p < .001. M = .60, SEM = .01$) and Test ($M = .64, SEM = .02, t(18) = 6.59, p < .001$) phase was significantly different than chance.

For participants best fit by the RL decision confusion model, there was a significant main effect of correct key press in both Learning ($F(1, 34) = 25.01p < .001$) and Test phase ($F(1, 34) = 15.05, p < .001$). There was no main effect of condition or interaction between condition and correct key press. In the Learning phase, there was a greater bias toward other rewarded keys in trials when the correct answer was 1 ($M = .74, SEM = .03$) than 2 ($M = .60, SEM = .01$), and both were significantly different than chance ($t(34) > 7.11, p < .001$). In the Test phase, both were significant prefer rewarded keys in other trials, but greater bias toward rewarded keys when correct answer was 1 ($M = .78, SEM = .04, t(10) = 7.44, p < .001$) than 2 ($M = .56, SEM = .02, t(10) = 3.03, p = .01$).

Model predictions do not successfully capture qualitative data patterns. Neither of the models are able to capture the avoidance of the unrewarded key in both phases, suggesting there is another process at work we did not include in the model. The RL decision confusion model is able to capture the qualitative effect of greater bias in "1" trials over "2" trials in Learning phase, but not in Test phase. Perhaps the RL decision confusion is able to capture greater bias in early learning, but stimulus confusion is lessened by late learning Q-values (which the test phase is based on).

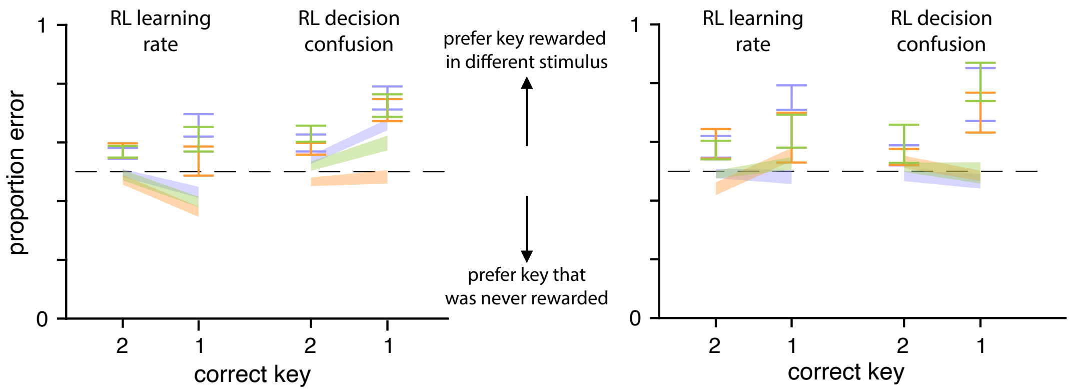

Figure S3: **Error types by winning model.** The proportion of incorrect key presses that were rewarded for other stimuli, based on how many stimuli shared the same key press (x-axis). Randomly responding between the two incorrect keys is shown with the dashed black line; above chance means a preference toward the key rewarded for a different stimulus. $M \pm SEM$ data (error bars) and model predictions (fills) for Learning (left; both experiments) and Test (right; Exp 2) phase.

S1.4 Parameter recovery

In order to establish the interpretability of model parameters, one should test that the same parameters that generate a data set are the ones recovered through the model parameter estimation method (Wilson & Collins, 2019). Successful parameter recovery exists when the parameter values that maximize the likelihood of the data given the model parameters are close to the parameter values that generated the data. Successful parameter recovery is necessary to interpret estimated parameter values.

For each model, we generated parameters by sampling the fitted parameter vectors from participants across both experiments. We sampled 50 participants without replacement. Our goal here was to use parameter values that best reflect the regime of the parameter space that matches data we are interested in. We also completed parameter recovery by sampling parameters from a nonparametric distribution informed by the fitted parameter values, rather than using the exact values. Because there are arbitrary decisions required to define this distribution, we did not include the results here. However, the results are qualitatively the same.

For each model and simulated participant, we simulated data with the sampled parameters, then estimated parameters using the same model fitting methods described in the main text. Finally, we plot the true and estimated parameters against one another. For each plot, values clustered along the diagonal indicate successful parameter recovery.

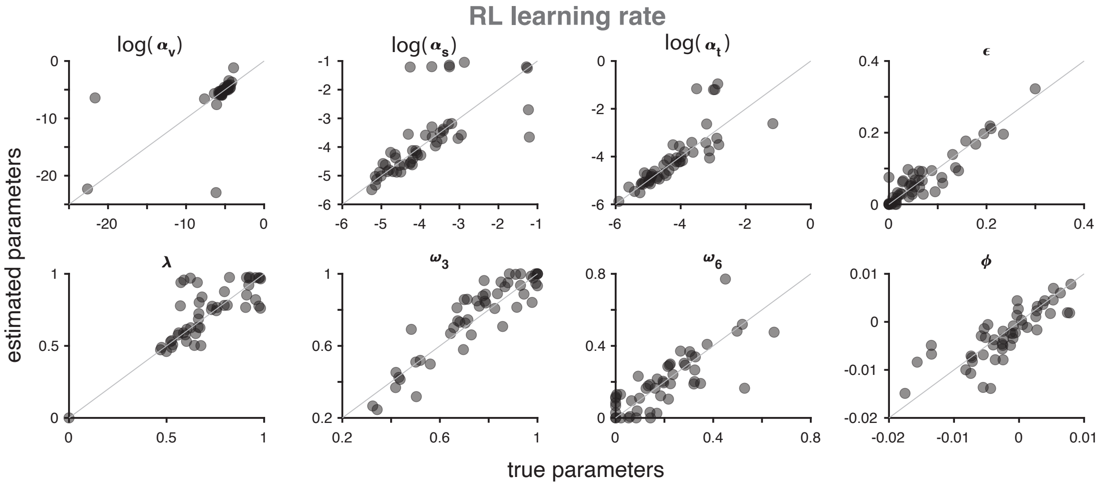

Figure S4: Parameter recovery plots for condition-specific RL learning rate model. Each subplot plots the true parameters (x-axis), which generated data, against the recovered parameter values (y-axis), estimated using MLE. Dots are individual simulated participants.

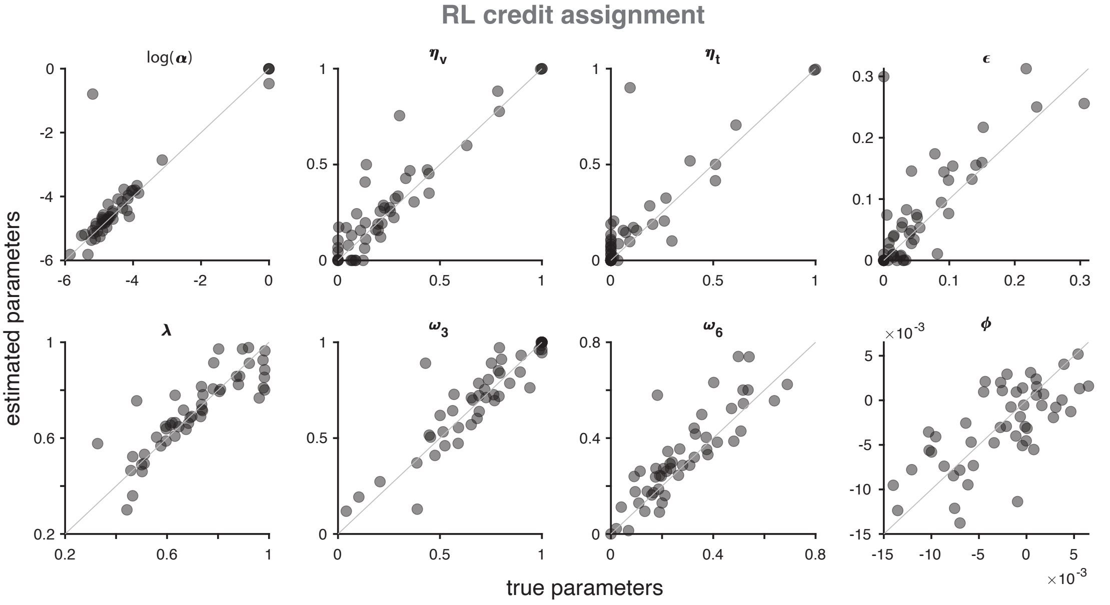

Figure S5: Parameter recovery plots for condition-specific RL credit assignment model. Each subplot plots the true parameters (x-axis), which generated data, against the recovered parameter values (y-axis), estimated using MLE. Dots are individual simulated participants.

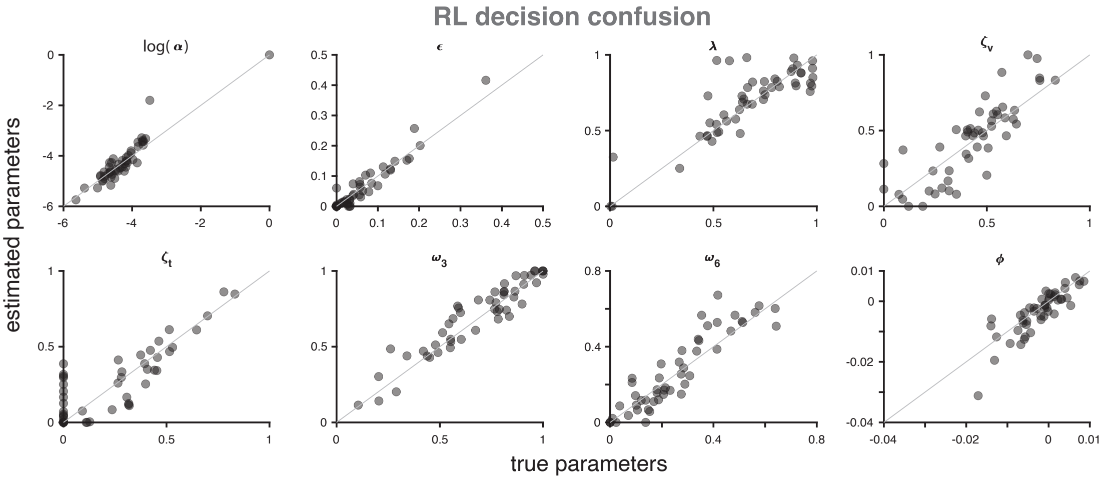

Figure S6: Parameter recovery plots for condition-specific RL decision confusion model. Each subplot plots the true parameters (x-axis), which generated data, against the recovered parameter values (y-axis), estimated using MLE. Dots are individual simulated participants.

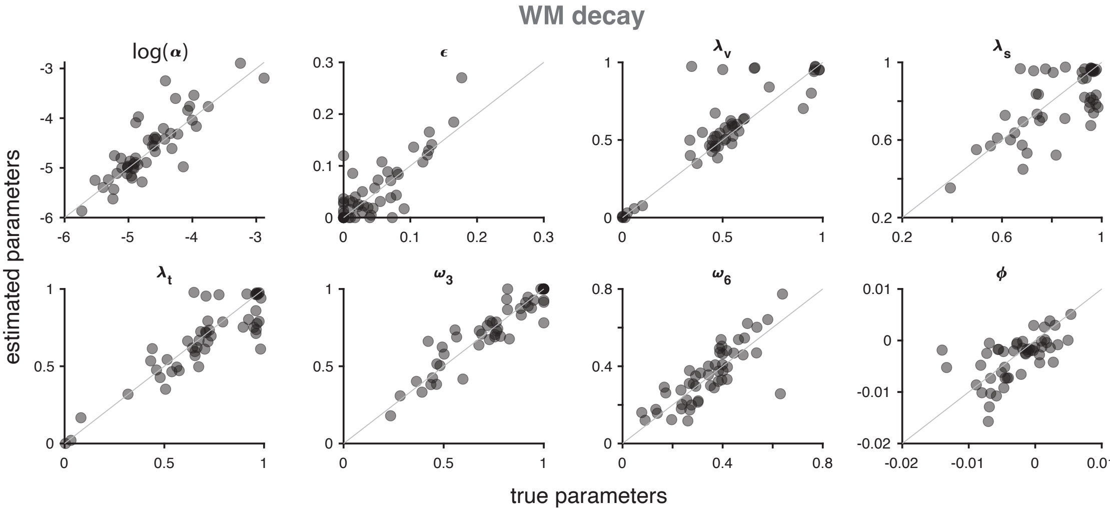

Figure S7: Parameter recovery plots for condition-specific WM decay model. Each subplot plots the true parameters (x-axis), which generated data, against the recovered parameter values (y-axis), estimated using MLE. Dots are individual simulated participants.

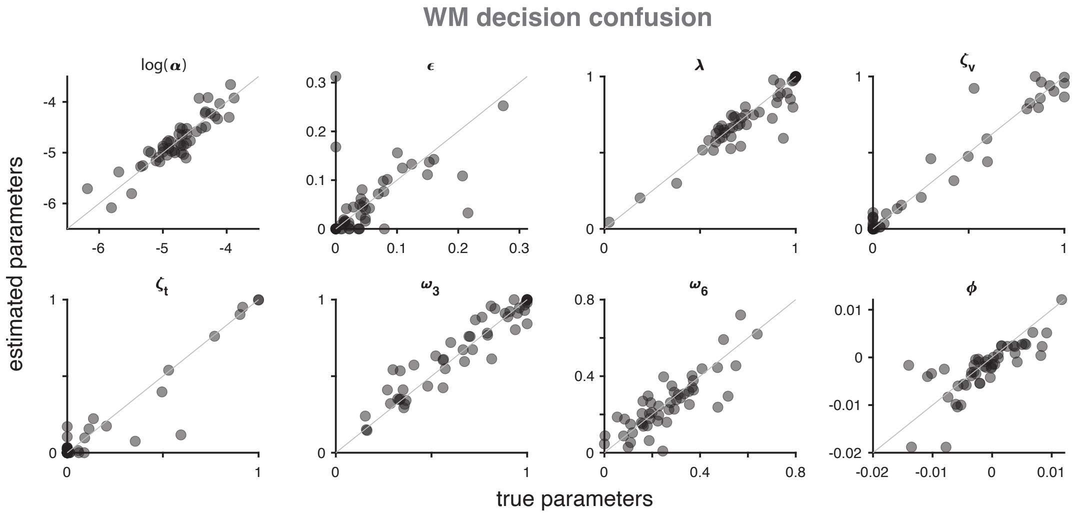

Figure S8: Parameter recovery plots for condition-specific WM decision confusion model. Each subplot plots the true parameters (x-axis), which generated data, against the recovered parameter values (y-axis), estimated using MLE. Dots are individual simulated participants.

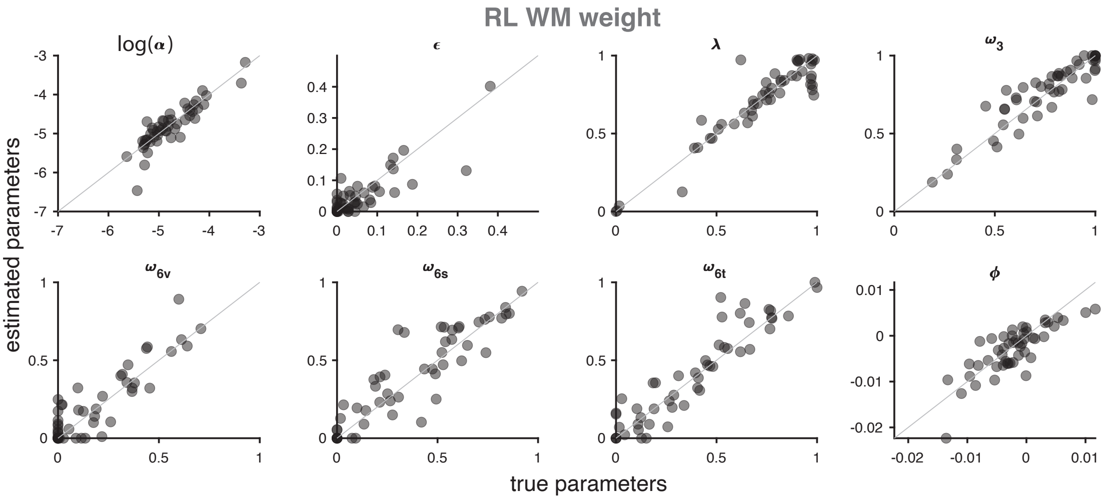

Figure S9: Parameter recovery plots for condition-specific RL WM weight model. Each subplot plots the true parameters (x-axis), which generated data, against the recovered parameter values (y-axis), estimated using MLE. Dots are individual simulated participants.

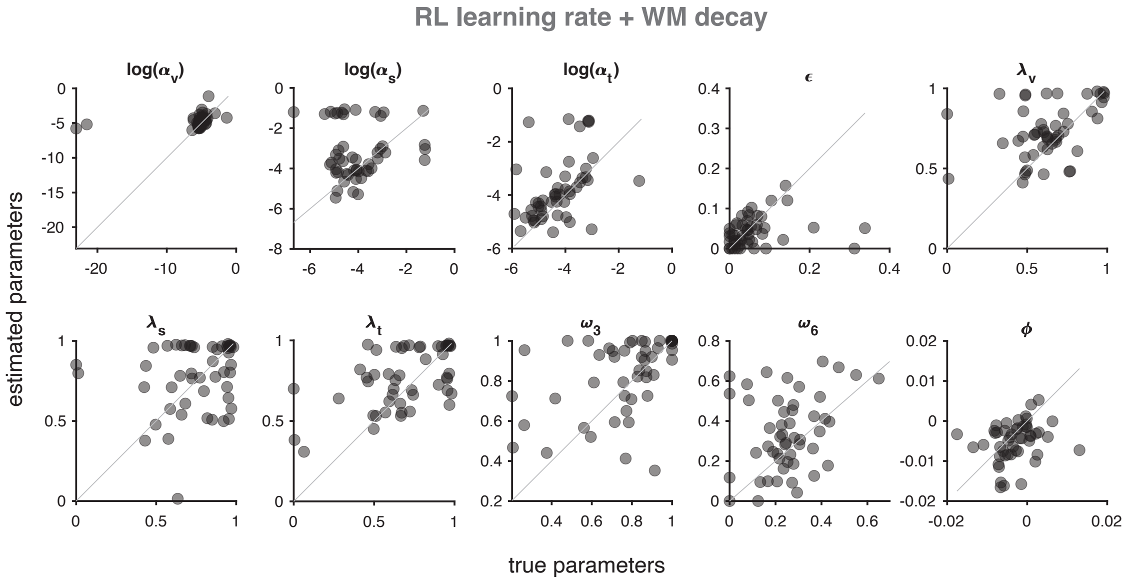

Figure S10: Parameter recovery plots for condition-specific RL learning rate + WM decay model. Each subplot plots the true parameters (x-axis), which generated data, against the recovered parameter values (y-axis), estimated using MLE. Dots are individual simulated participants.

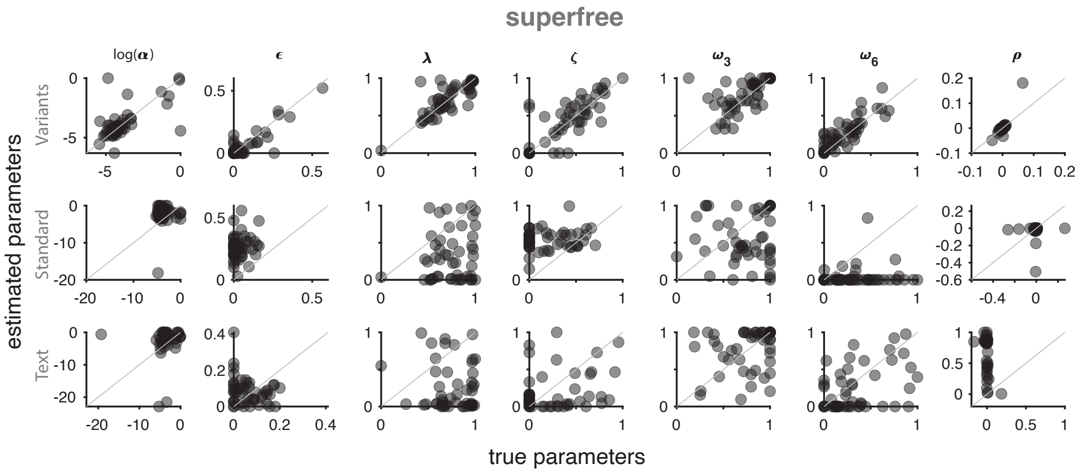

Figure S11: Parameter recovery plots for superfree model. Each subplot plots the true parameters (x-axis), which generated data, against the recovered parameter values (y-axis), estimated using MLE. Dots are individual simulated participants.

S1.5 Model recovery

Model recovery is an important step before making conclusions from a quantitative model comparison (Wilson & Collins, 2019). Successful model recovery occurs when the same model that generates a data set best fits it (according to your chosen model comparison metrics), when compared to all other models in the comparison set.

For each model, we generated 50 simulated participants' data from the parameter values fitted from 50 participants, randomly sampled without replacement from both experiments. (We use the same simulated participants' data for parameter recovery). We then fit every model to each of of these (nModels x 50) simulated participants, using the same fitting methods as described in the main text.

We compared model goodness-of-fit using corrected Akaike Information Criterion (AICc), Bayesian Information Criterion (BIC), and $\exp_r$. AICc and BIC both penalize models with more parameters, and BIC penalizes more strictly:

$$\text{AICc} = -2LL^* + 2k + \frac{2k(k+1)}{N_{\text{trials}} - k - 1}$$

$$\text{BIC} = -2LL^* + k \log N_{\text{trials}},$$

where k is the number of parameter and N_{trials} is the number of trials.

The measure exp_r is calculated using BMS spm, which explicitly assumes that the participants can be fit by different models. This value is expectation of the posterior probabilities of each model.

Successful model recovery occurs when the model that best fits a simulated data set is the same model that generated that data set. For example, if all 50 participants generated by the condition-specific RL learning rate model are best fit by the condition-specific RL learning rate model, there is successful model recovery.

For the most part, we consider our results successful model recovery (Figure S12). However, these results also indicate the RL learning rate, WM decay, and RL WM weight models are a bit more flexible than others, demonstrated by their ability to best capture data sets generated from other models. These results suggest that model comparisons favoring these three models may be do to model flexibility, rather than a genuine reflection of the underlying cognitive process. In our experimental data (see main manuscript), we do indeed find that the RL learning rate model fits the data best. However, because 1) we do not find that WM decay or RL WM weight models fit the data as well, and 2) the RL decision confusion model is able to fit the data comparably well to the RL learning rate model, we believe our interpretation of the results (i.e., that RL is specifically affected, but not committing to how) is still valid.

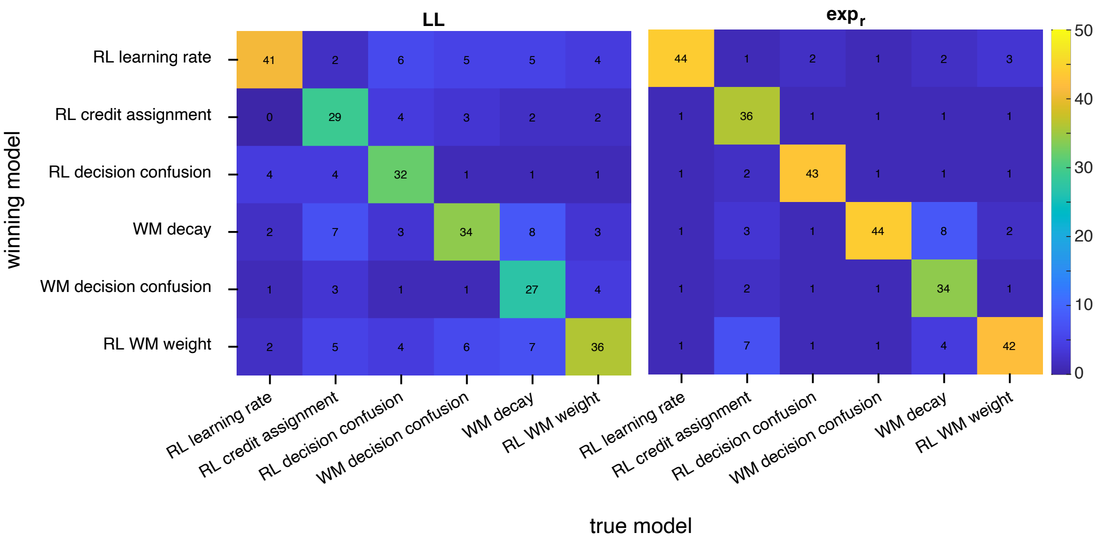

Figure S12: Model recovery when using LL^* and expected probability using BMS SPM ($\exp_r$), for six main models with same number of parameters. Successful model recovery is indicated by a majority of models falling on the diagonal. Both metrics provide good model recovery, although $\exp_r$ is a bit better.

Our model comparison including the additional two models (RL learning rate + WM decay, superfree) are not as simple, due to the relatively high confuseability of the RL learning rate model and the RL learning rate + WM decay model (Figure S13). We did an additional model recovery analysis between just these two models, with 500 simulated datasets, 50 parameter sets each simulated 10 times (Figure S14). Although the majority tends in the desired direction, the simpler RL learning rate model is able to account for much of the more complex RL learning rate + WM decay model. Thus, our model comparison results between these two models should be taken with a grain of salt.

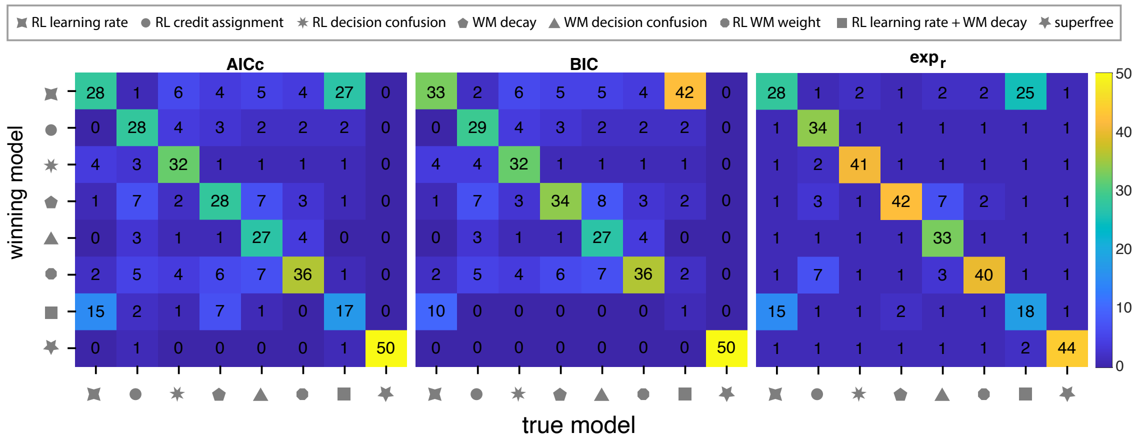

Figure S13: Model recovery when using AICc, BIC, and expected probability using BMS SPM ($\exp_r$). Successful model recovery is indicated by a majority of models falling on the diagonal. These results generally convey reasonable model recovery, for all models except the RL learning rate + WM decay model. AICc and $\exp_r$ provide better recovery than BIC.

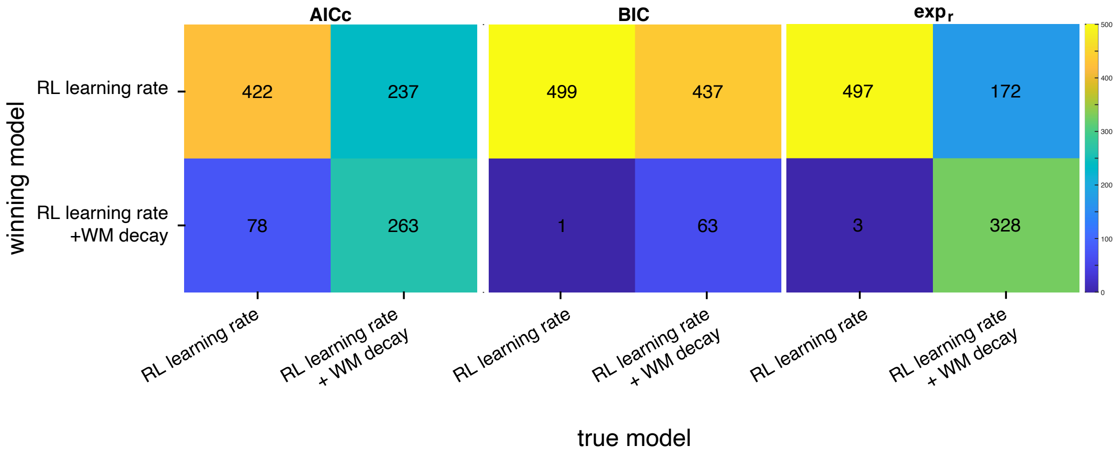

Figure S14: A follow up model recovery with more simulated data (independent from earlier datasets), with just the "RL learning rate" and "RL learning rate + WM decay" models, which had the greatest confusability in earlier model recovery plots. No metric is able to capture a desired level of model recovery, although AICc and $\exp_r$ are able to capture the correct directionality.

S1.6 Parameter values

In this section, we plot the individual and group parameter values for the two winning models: the condition-specific RL learning rate model (Figure S15) and condition-specific RL decision confusion model (Figure S16).

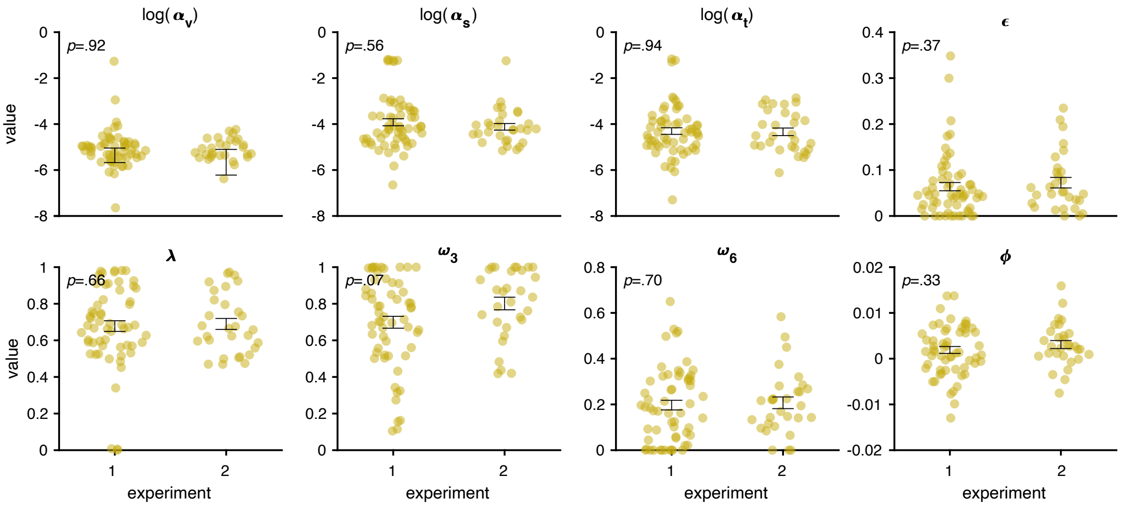

Figure S15: Parameter values (dots: individual participants. error bars: $M \pm sem$ across participants) for the condition-specific RL learning rate model for Experiment 1 and Experiment 2. Outliers for $\log(\alpha_v)$ not illustrated in plot (Exp 1: -21.66; Exp 2: 22.63). The p-values of a Wilcoxon rank sum test comparing the two participant groups, *before* any multiple comparisons corrections, displayed on the top left of each subplot.

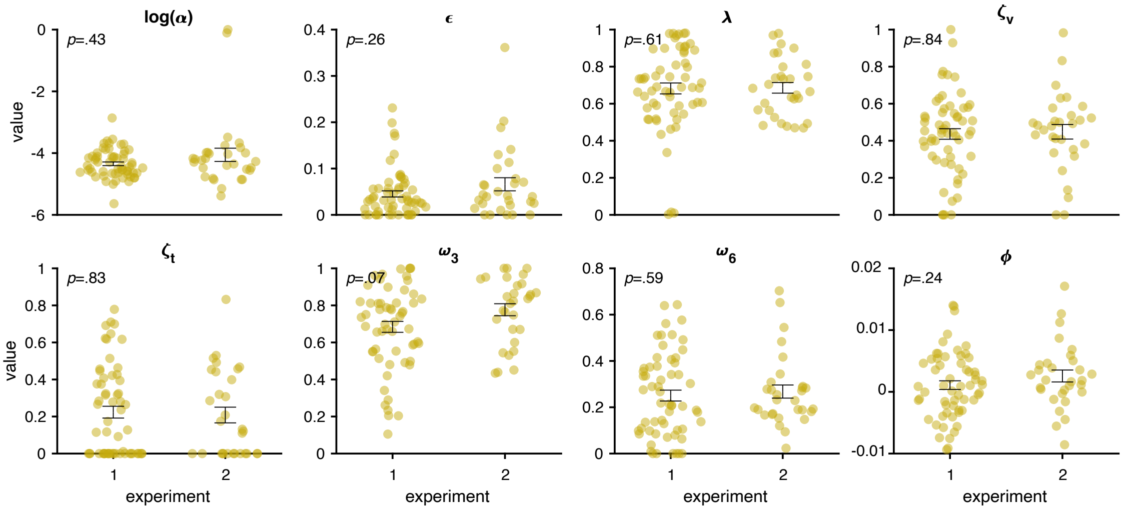

Figure S16: Parameter values (dots: individual participants. error bars: $M \pm sem$ across participants) for condition-specific RL decision confusion model for Experiment 1 and Experiment 2. The p-values of a Wilcoxon rank sum test comparing the two participant groups, *before* any multiple comparisons corrections, displayed on top left of each subplot.

S1.7 Alternative Models

We tested six main models in the manuscript with the following condition-specific differences: RL learning rate, RL credit assignment, RL decision confusion, WM decay, WM decision confusion,

and weight between RL and WM process contributions. There are of course an infinite amount of other models that we could have tested. This section summarizes related models that we fitted, that may be of interested to the reader. We divide this section into three parts. First, we display the results of models with only an RL component, only a WM component, and standard RLWM models without condition-dependencies. These models are common to report in similar studies, but were not reported in our main manuscript because they are obviously poorly fitting models. Second, we use factorial model comparison to test whether the goodness of fit for the eight main models we fit in the main manuscript vary with/without perseveration, and with/without a fitted negative learning rate, α_-, parameter. There are published studies suggesting the assumptions we included in the main manuscript were reasonable, but we still chose to test them directly. Third, we test if our assumption of 1-back perseveration (i.e., the time decay of perseveration) affects our modeling results, by softening this assumption. Fourth, we show model validation plots for the additional models considered in the main manuscript: the RL learning rate + WM decay model and the Superfree model. Finally, we show model validation plots for the additional models considered in Experiment 2: the RL learning rate and RL decision confusion models with condition-specific interference of WM on RL during learning.

In these sections, we compared model goodness-of-fit using AICc and BIC.

S1.7.1 RL, WM, RLWM model fits

Three models that are often shown in "RLWM" papers are RL alone, WM alone, and RL+WM models. We decided not to show their fits in the main manuscript, because they explicitly do not include any condition-specific differences, and would thus obviously not fit the data well. However, for the sake of completeness and comparison, we include the model validation and model comparison plots of Experiment 1 participants, relative to the condition-specific RL learning rate model used in the main manuscript. Indeed, they are not able to capture the data (Figure S17).

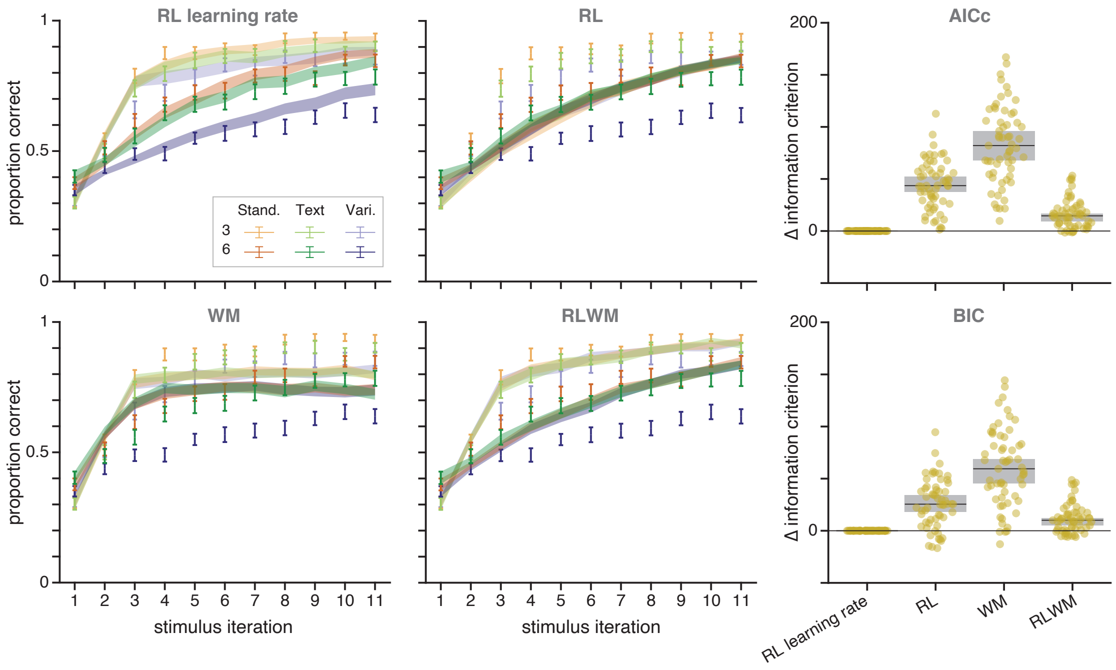

Figure S17: Model validation plots for the condition-specific RL learning rate, RL, WM, and RLWM models (left four plots) for Experiment 1 data. AICc (top) and BIC (bottom) differences between models and RL learning rate model. A smaller number indicates a better fit. The condition-specific RL learning rate clearly fit the data qualitatively and quantitatively better than these models.

S1.7.2 Perseveration and negative learning rate

In our main six models, we fit a perseveration rate ϕ, and we fix negative learning rate α_- to 0. Here, we factorially compare model family (6: RL learning rate, RL credit assignment, RL decision confusion, WM decay, WM decision confusion, and RL-WM weight), perseveration (2: fixed to 0, fit as free parameter), and negative learning rate (2: fixed to 0, fit as free parameter).

Figure S18 illustrates the quantitiative comparison of all models for both AICc and BIC. We find that fitting a perseveration parameter does seem to increase the model's quantitative fit, while fitting a negative learning rate parameter does not seem to make a difference. (This is because the values are fit to 0). More importantly, we see that the ranking across model family doesn't vary no matter what perseveration / negative learning rate combination we use. In other words, our conclusion that RL learning rate and RL decision confusion models fit data best are not dependent on our specific assumptions about perseveration or negative learning rate. For simplicity, we decided in the main manuscript to include the model which keeps perseveration as a free parameter, and fixed negative learning rate $\alpha_- = 0$.

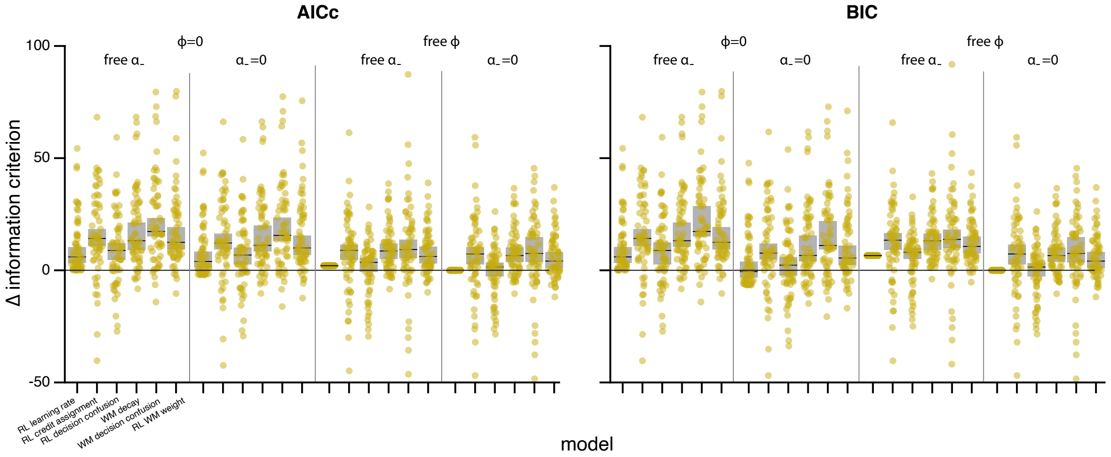

Figure S18: Quantitative results of factorial model comparison. AICc (left) and BIC (right) differences, relative to the RL learning rate model in the main manuscript. A lower number indicates a better fit. For each plot, each section of six models correspond to the respective characteristics: $\phi = 0$, fitted α_-; $\phi = 0$, $\alpha_- = 0$; fitted ϕ and α_-; fitted ϕ, $\alpha_- = 0$

S1.7.3 Perseveration with free decay rate parameter

We define perseveration in the Modeling Methods section of the main manuscript, in which we fix the perseveration choice trace decay rate, τ, to 1. Thus, only the previous trial affects the current perseveration behavior. We investigate in this section whether that was a reasonable assumption, by fitting the decay rate τ as a free parameter. Freeing this parameter neither significantly increases model performance of any of our main six models nor changes model ranking.

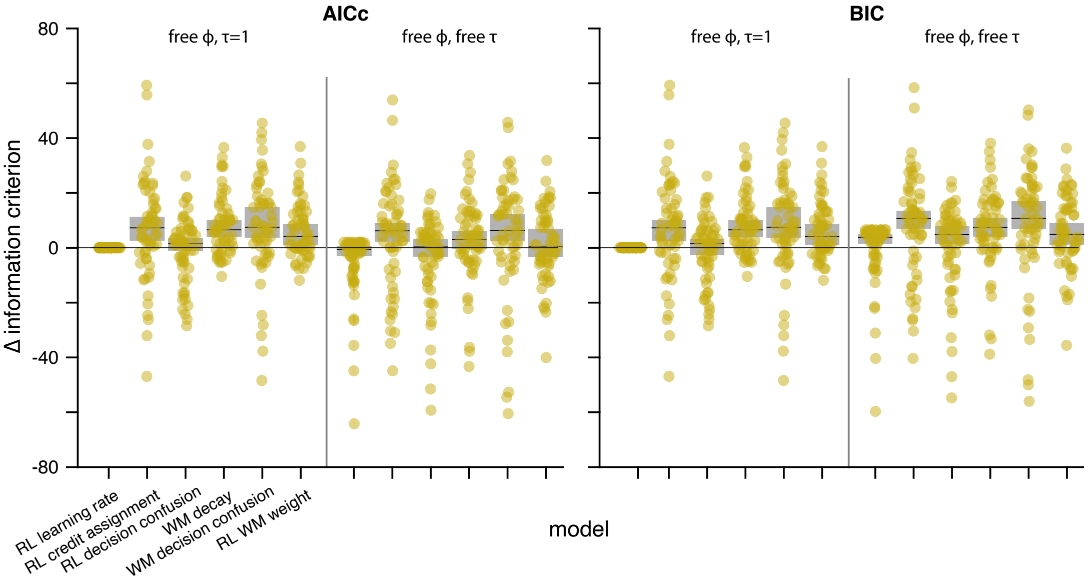

Figure S19: Factorial model comparison with perseveration parameter τ fixed to 1 (left six models on each plot) and as a free parameter (right six models on each plot). AICc (left plot) and BIC (right plot) are relative to the RL learning rate model with $\tau = 1$. A lower value indicates a better fit to data. Model differences do not change model rankings, and model fits are not noticeably improved by including a free τ parameter.

S1.7.4 RL learning rate + WM decay model, Superfree model

In this section, we show the model validation and model comparison plots for the two additional models considered in the main manuscript.

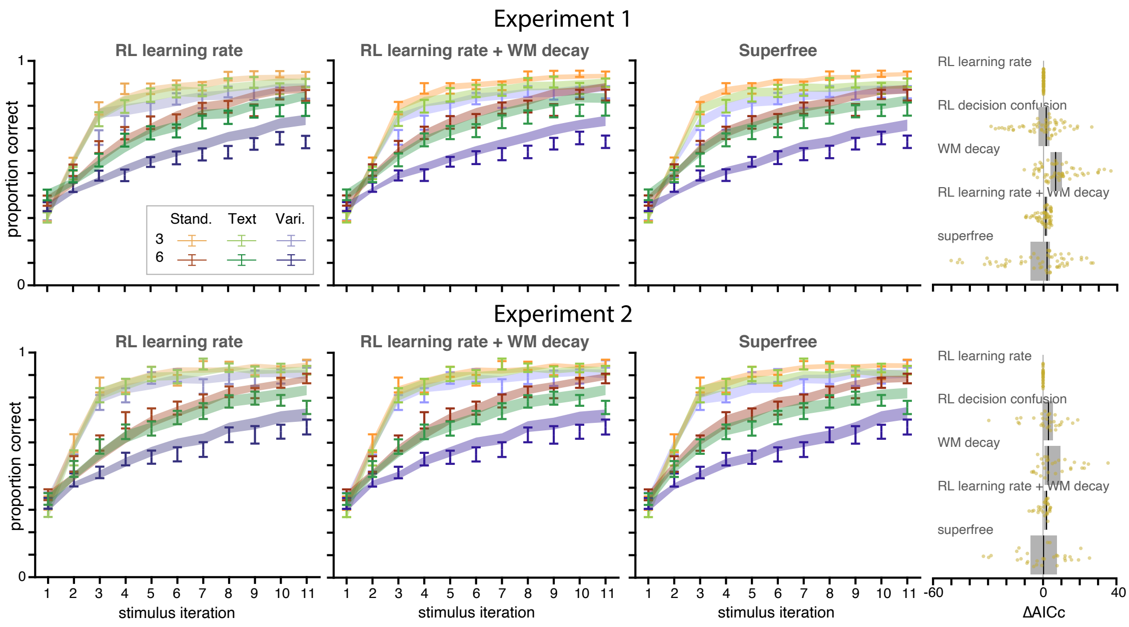

Figure S20: Model validations of RL learning rate + WM decay model and Superfree model for Experiment 1 (top row) and Experiment 2 (bottom row). We plot them next to the model validation of the RL learning rate model, which is our best fitting model. We show quantitiavie model comparison for each participant (yellow dots), with bootstrapped median 90 CI of the median (grey box). All other quantiative model comparison metrics are displayed in tables 2 and 3 in the main text.

S1.7.5 Condition-specific interaction for train+test models

In this section, we describe models that were fitted with different degrees of RL/WM interference between train and test in different conditions.

The δ used in updating Q values in interference model includes the WM values, rather than just Q values (Eq. 2). For condition-specific interference, we additionally add a multipllicative term to scale the amount of interference the WM value association gives when calculating delta. We denote the condition-specific interference scalar as x_c for condition c.

$$\delta = r - (\omega_n x_c * WM(s,a) + (1 - \omega_n x_c) * Q(s,a)).$$

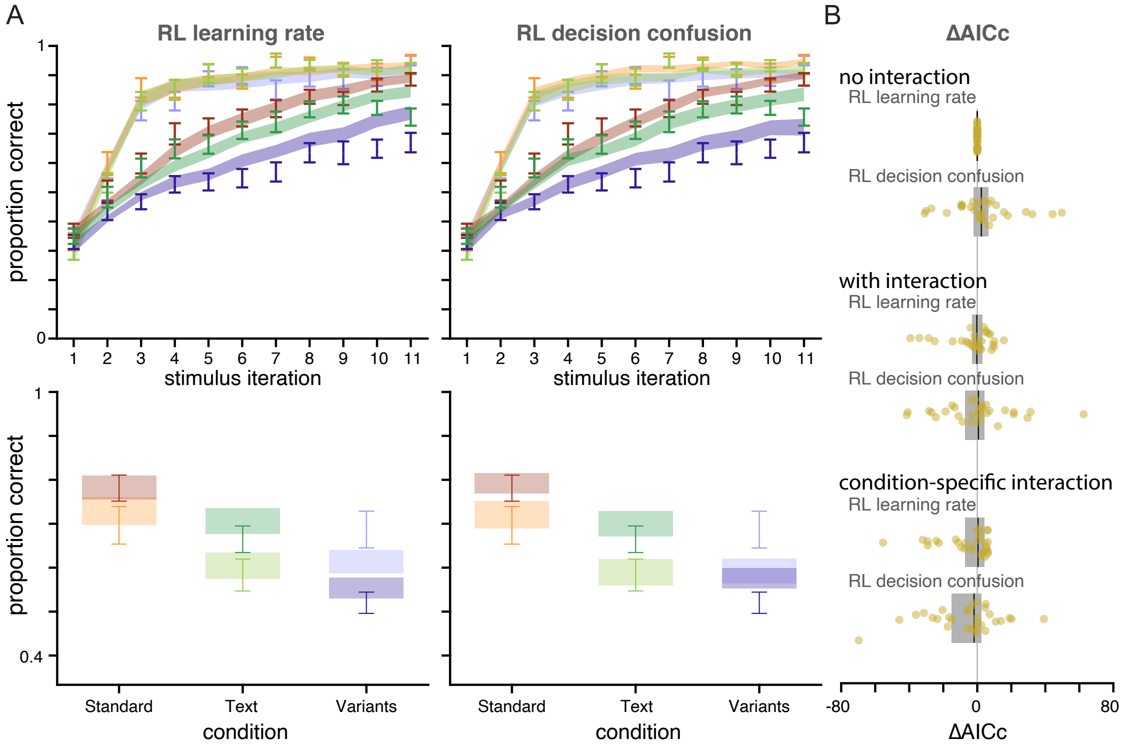

Figure S21: **Model validation and comparison for condition-specific interference models. A**. Model validation for RL learning rate (left plots) and RL decision confustion (right plots) model with condition-specific interference. Top row corresponds to learning phase, bottom row corresponds to test phase behavior (error bars) and model predictions (color fill). **B.** AICc differences of all models fit on learning and test phase data, relative to RL learning rate model with no interference. Negative values indicate better fit. Including condition-specific interference (last two) marginally improves fit, but still does not capture data perfectly.

References

Wilson, R. C., & Collins, A. G. (2019). Ten simple rules for the computational modeling of behavioral data. *eLife*, *8*, e49547. doi: 10.7554/eLife.49547
